# Cytosolic PRDX1 acts as an extramitochondrial sink to set mitochondrial H_2_O_2_ levels and enable resilience to chronic mitochondrial oxidative stress

**DOI:** 10.1016/j.redox.2026.104195

**Published:** 2026-04-30

**Authors:** Lianne JHC. Jacobs, Sebastian Doll, Dietrich Trümbach, Matteo Veronese, Giada Di Pietro, Fatma Isil Yapici, Lidwina Hasberg, Pascal Gentzsch, Sarah Gerlich, Jens Hansen, Silvia von Karstedt, Elena I. Rugarli, Marcus Conrad, Armindo Salvador, Jan Riemer

**Affiliations:** a Redox Metabolism Group, Institute for Biochemistry, University of Cologne, Cologne, 50674, Germany; b Cologne Excellence Cluster on Cellular Stress Responses in Aging-Associated Diseases (CECAD), University of Cologne, Cologne, 50931, Germany; c Helmholtz Zentrum München, Institute of Metabolism and Cell Death, Neuherberg, 85764, Germany; d Institute for Genetics, University of Cologne, Cologne, 50674, Germany; e Department of Translational Genomics, Faculty of Medicine and University Hospital Cologne, University of Cologne, Cologne, 50931, Germany; f Center for Molecular Medicine, University of Cologne, 50931 Germany; g Translational Redox Biology, Technical University of Munich (TUM), TUM Natural School of Sciences, Garching, 85748, Germany; h CNC-UC - Centre for Neuroscience Cell Biology, University of Coimbra, Coimbra, 3004-504, Portugal; i CiBB - Centre for Innovative Biomedicine and Biotechnology, University of Coimbra, Coimbra, 3004-504, Portugal; j Coimbra Chemistry Center ‐ Institute of Molecular Sciences (CQC‐IMS), University of Coimbra, Coimbra, 3004-535, Portugal; k Institute for Interdisciplinary Research, University of Coimbra, Coimbra, 3030-789, Portugal

## Abstract

Hydrogen peroxide (H_2_O_2_) plays a dual role as both a signalling molecule and a mediator of oxidative stress. Although mitochondria are major producers of H_2_O_2_, the relative contributions of mitochondrial versus cytosolic antioxidant systems to mitochondrial H_2_O_2_ homeostasis in intact cells remain poorly defined. Here, we combined compartment-resolved live-cell imaging using HyPer7, inducible mitochondrial H_2_O_2_ generation (matrix-targeted d-amino acid oxidase), kinetic modelling, and a targeted CRISPR/Cas9 screen to dissect determinants of mitochondrial H_2_O_2_ dynamics in HEK293 cells. Unexpectedly, we found that the cytosolic peroxiredoxin PRDX1 is a dominant regulator of mitochondrial matrix H_2_O_2_ levels. Loss of cytosolic PRDXs markedly enhanced matrix Hyper7 signals under both exogenous and mitochondria-intrinsic H_2_O_2_ production, exceeding the effects of deleting mitochondrial peroxiredoxins. Modelling and transport experiments indicated a very high permeability of the mitochondrial inner membrane to H_2_O_2_ enabling rapid efflux and the establishment of steep concentration gradients. This permits the cytosol to function as a major sink to limit matrix H_2_O_2_ accumulation. PRDX1 deficiency sensitized cells to chronic mitochondrial oxidative stress. A targeted CRISPR screen identified the Rab7 GAP TBC1D5, linking mitophagy to cellular survival under these conditions. Consistently, PRDX1/2-deficient cells exhibited elevated mitophagic flux, indicating mitochondrial quality control as a compensatory response. Our study reveals that cytosolic PRDXs critically impact mitochondrial redox homeostasis and provides a systems-level framework for understanding compartmental redox control and stress adaptation.

## Highlights

• Cytosolic PRDX1 is a dominant regulator of mitochondrial matrix H_2_O_2_ levels in intact cells.
• High inner mitochondrial membrane permeability enables cytosolic scavenging of matrix H_2_O_2_.
• Cytosol functions as a major sink shaping mitochondrial redox homeostasis.
• TBC1D5-dependent mitophagy supports survival under chronic mitochondrial oxidative stress.

## Graphical abstract

**Figure:**
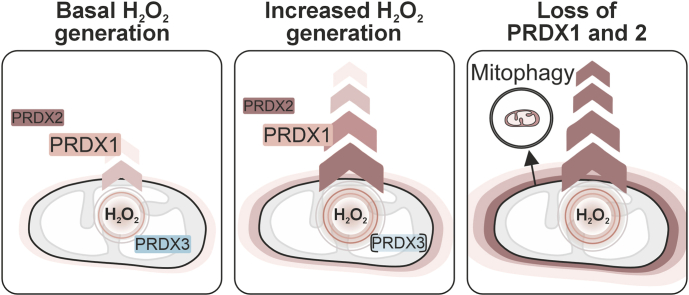

## 1 Introduction

Reactive oxygen species (ROS) are reactive molecules derived from oxygen, including free radicals such as superoxide and hydroxyl radicals, as well as non-radical molecules like hydrogen peroxide (H_2_O_2_), a relatively stable ROS that can diffuse across cell membranes. Traditionally ROS was associated with oxidative damage to lipids, proteins, and DNA and therefore implicated in aging and numerous pathologies. However, ROS, and in particular H_2_O_2_, are currently also recognized as critical signaling molecules in a wide range of physiological and stress-related processes including autophagy, immune responses, and cellular differentiation [[1], [2], [3], [4], [5], [6], [7], [8], [9], [10]].

Because of their double-edged role, the spatial and temporal dynamics of H_2_O_2_ need to be tightly regulated. Local H_2_O_2_ levels are thereby set by a balance between generators, such as NADPH oxidases, metabolic dehydrogenases, and the mitochondrial respiratory chain, and scavenging systems, such as catalase, and glutathione or thioredoxin peroxidases (GPX, PRDX) [1,8,11]. Moreover, diffusion out of the respective compartment also contributes to lowering local H_2_O_2_ levels [[12], [13], [14], [15], [16]]. Antioxidative systems are thought to remove >99% of the cellular H_2_O_2_ before it can interact with other biomolecules [17]. However, under certain physiological conditions H_2_O_2_ formation can occur at relatively high rates [18], and cellular H_2_O_2_ concentrations can reach deleterious levels of >1 μM that lead to oxidative damage of different biomolecules and may result in reduced cell proliferation and cell death [19].

Mitochondria are both a primary source and a target of H_2_O_2_. Respiratory chain complexes I, II and III as well as metabolic dehydrogenases in the matrix contribute to mitochondrial H_2_O_2_ generation [1,11,[20], [21], [22]]. Mitochondria contain a set of antioxidative enzymes that are distinct from their cytosolic counterparts, *e.g.* while the cytosol contains the 2-Cys PRDX1 and 2, the matrix harbors the 2-Cys PRDX3 and 5 [23]. Maintaining mitochondrial redox homeostasis not only involves handling of H_2_O_2_ by antioxidative systems, but also involves dealing with the consequences of redox disbalances. For example, mitochondrial DNA that is susceptible to oxidative damage can be repaired, or transcriptional regulation by the NRF2-KEAP1 axis might allow mitochondrial import of antioxidative factors. Upon mitochondrial dysfunction, damaged mitochondria are selectively cleared by the selective autophagy of mitochondria (mitophagy) [24]. Mitochondrial dynamics can also contribute to this process as fusion allows mixing of mitochondrial contents to dilute damage, while fission isolates damaged sections for removal via mitophagy [[24], [25], [26], [27]].

The contribution of 2-Cys PRDXs to mitochondrial H_2_O_2_ dynamics is still poorly understood as it depends on many different factors including expression levels, enzymatic activity, occurrence of competing oxidative influences, availability of reducing equivalents and machineries, and the diffusion rates of H_2_O_2_ across the mitochondrial membranes. We performed here a systematic experimental and modelling analysis of the contribution of the different cytosolic and mitochondrial PRDXs in intact HEK293 cells. We thereby found cytosolic PRDX1 to be the major contributor not only to H_2_O_2_ dynamics in the cytosol, but also to cellular resilience towards mitochondria-generated H_2_O_2_. Moreover, we demonstrated that PRDX1 becomes the major contributor to H_2_O_2_ dynamics in the mitochondrial matrix when this compartment is exposed to pathophysiological H_2_O_2_ generation rates. Steady state H_2_O_2_ measurements and modeling approaches indicate that this can also happen at physiological H_2_O_2_ generation rates that do not overwhelm the local reductive capacity since mitochondrial membranes seem to allow efficient H_2_O_2_ release due to very high H_2_O_2_ permeabilities. We then employed a CRISPR/Cas9 drop-out screen to determine further contributors to sustaining mitochondrial H_2_O_2_ stress in PRDX1/2 DKO cells. We thereby found the Rab7 GAP TBC1D5 as a major hit, which has been implicated in controlling mitophagy. In line, we found mitophagic flux to be increased in PRDX1/2 DKO cells likely as compensatory mechanism.

## 2 Results

### 2.1 PRDX1 exerts the main control on mitochondrial matrix H_2_O_2_ levels and dynamics upon treatments with exogenous H_2_O_2_ bolus

**Fig. 1:**
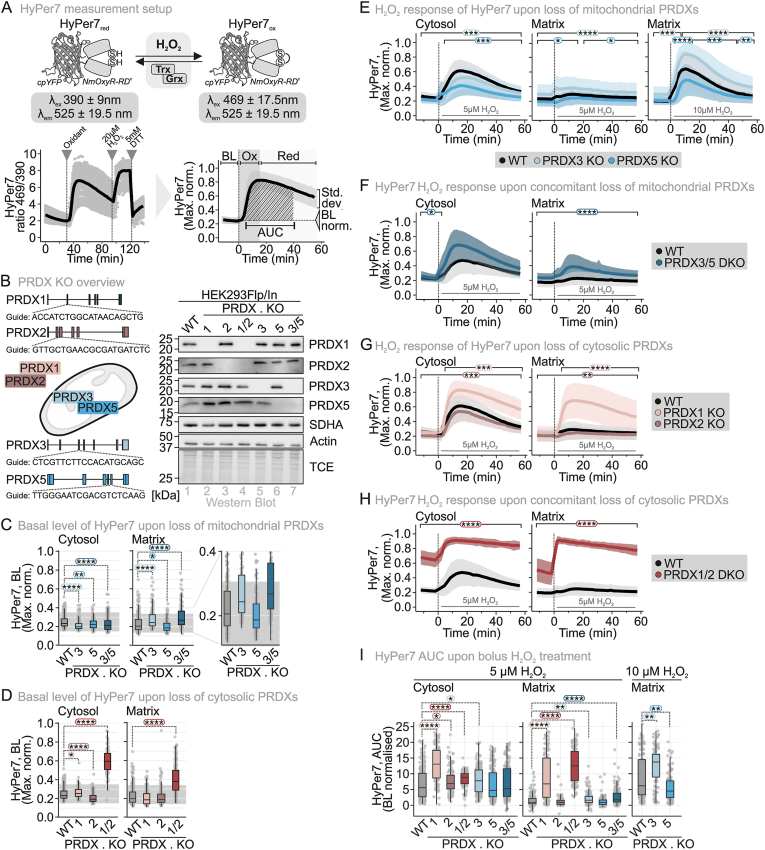
**PRDX1 is the main determinant of mitochondrial matrix H_2_O_2_ levels and dynamics upon treatments with exogenous H_2_O_2_** (A) HyPer7 measurement setup and representation of data. Data is normalized to the maximum oxidation (Max. norm.) of each cell. The base level (BL) is calculated as average over a 10-min timespan (−10 to 0 min), and the AUC (area under the curve) is calculated over a 35-min timespan (5 to 40 min) by subtracting the BL. OX: oxidation, Red: reducing. (B) Peroxiredoxin levels in the generated single and double knockout (KO, DKO) cell lines. *Left*: guides used to generate the KO cell lines. *Right*: immunoblot analysis to confirm the generated KO cell lines. (C) Basal level (BL) of HyPer7 upon loss of mitochondrial PRDXs. HyPer7 sensors were targeted to the indicated subcellular compartments. Cells were grown in glucose-containing medium. The grey area indicates a deflection of 0.1 from the BL of the WT. The number of cells, number of experimental replicates and information on statistical analysis and visualization can be found in **Supplementary dataset S1**. (D) Basal level (BL) of HyPer7 upon loss of cytosolic PRDXs. HyPer7 sensors were targeted to the indicated subcellular compartments. Cells were grown in glucose-containing medium. The number of cells, number of experimental replicates and information on statistical analysis and visualization can be found in **Supplementary dataset S1**. (E) Response of HyPer7 in mitochondrial PRDX KO cell lines upon treatment with 5 μM extracellular H_2_O_2_ (black, wild type; light blue, PRDX3 KO; blue, PRDX5 KO). HyPer7 sensors were targeted to the indicated subcellular compartments. The cells were grown in glucose-containing medium. Solid line represents average, and the standard deviation is represented by the background in the respective color. The number of cells, number of experimental replicates and information on statistical analysis and visualization can be found in **Supplementary dataset S1**. (F) Response of HyPer7 in mitochondrial PRDX DKO cell line upon treatment with 5 μM extracellular H_2_O_2_ (black, wild type; dark blue, PRDX3/5 DKO). HyPer7 sensors were targeted to the indicated subcellular compartments. The cells were grown in glucose-containing medium. Solid line represents average, and the standard deviation is represented by the background in the respective color. The number of cells, number of experimental replicates and information on statistical analysis and visualization can be found in **Supplementary dataset S1**. (G) Response of HyPer7 in cytosolic PRDX KO cell lines upon treatment with 5 μM extracellular H_2_O_2_ (black, wild type; pink, PRDX1 KO; brown, PRDX2 KO). HyPer7 sensors were targeted to the indicated subcellular compartments. The cells were grown in glucose-containing medium. Solid line represents average, and the standard deviation is represented by the background in the respective color. The number of cells, number of experimental replicates and information on statistical analysis and visualization can be found in **Supplementary dataset S1**. (H) Response of HyPer7 in cytosolic PRDX DKO cell line upon treatment with 5 μM extracellular H_2_O_2_ (black, wild type; dark red, PRDX1/2 DKO). HyPer7 sensors were targeted to the indicated subcellular compartments. The cells were grown in glucose-containing medium. Solid line represents average, and the standard deviation is represented by the background in the respective color. The number of cells, number of experimental replicates and information on statistical analysis and visualization can be found in **Supplementary dataset S1**. (I) AUC of the HyPer7 oxidation upon treatment with 5 or 10 μM extracellular H_2_O_2_ in PRDX KO cell lines. HyPer7 sensors were targeted to the indicated subcellular compartments. Cells were grown in glucose-containing medium. The number of cells, number of experimental replicates and information on statistical analysis and visualization can be found in **Supplementary dataset S1**. ∗P ≤ 0.05, ∗∗P ≤ 0.01, ∗∗∗P ≤ 0.001, ∗∗∗∗P ≤ 0.0001.

The dynamics of H_2_O_2_ within mitochondria, the influence of the cytosol, and the interplay of factors regulating mitochondrial H_2_O_2_ levels remain poorly understood. To address this, we utilized the genetically-encoded fluorescent probe HyPer7, which enables real-time monitoring of basal H_2_O_2_ levels in specific subcellular compartments [14]. HyPer7 is composed of a circularly permuted yellow fluorescent protein (cpYFP) inserted within the OxyR regulatory domain (OxyR-RD) of *Neisseria meningitidis* (Fig. 1A). The OxyR moiety directly reacts with H_2_O_2_, leading to disulfide bond formation, which in turn induces a shift in the fluorescence excitation spectrum of cpYFP. While the probe is oxidized by H_2_O_2_, it appears to be predominantly re-reduced by thioredoxins in the cytosol [28] and by the glutathione system in the mitochondrial matrix [29]. Thus, the HyPer7 redox state is determined by the balance between rapid H_2_O_2_-driven oxidation and slower thioredoxin- or glutathione-mediated reduction (Fig. 1A). In our experimental setup, we monitored individual cells and quantified HyPer7 oxidation using a ratiometric fluorescence readout [30]. The signal was measured as the maximum-normalized ratio of fluorescence emission intensity following excitation at 469 nm and 390 nm, ensuring automatic compensation for variations in probe concentration. An increase or decrease in the maximum-normalized 469/390 ratio (Max. norm.) corresponds to increased or decreased average probe oxidation, respectively. Notably, HyPer7's ratiometric readout remains relatively stable across large pH shifts (pH 6 to 8) making it suitable for measurements in different compartments. Throughout this study, we report HyPer7 response curves over time alongside steady states or basal levels (BL) of HyPer7 and “area under the curve” (AUC) values (35 min of treatments, BL subtracted) (Fig. 1A). AUC allows for rapid comparison of cell behaviour across different cells and treatments.

To characterize the mitochondrial and cytosolic H_2_O_2_ dynamics, we generated HEK293 cells lacking key H_2_O_2_-scavenging proteins: the cytosolic peroxiredoxins (PRDX) 1 and 2 or the mitochondrial PRDX3 and 5 (Fig. 1B). In all single knockout (KO) and double KO (DKO) cell lines, levels of proteins summarized under the GO term “redox homeostasis” remained mostly unchanged when compared to wild type (WT) cells (Fig. S1A). Proliferation of KO and DKO cells was comparable to WT (Fig. S1B). PRDX5 KO cells appeared to have a slightly enhanced proliferation rate, which became more pronounced when shifted to galactose (Fig. S1B). Cellular glutathione levels remained unchanged upon loss of any of the PRDXs (Fig. S1C). Collectively, this indicates that HEK293 cells are able to compensate for the loss of PRDXs in both compartments under unperturbed conditions.

We then assessed HyPer7 BL-ratios in PRDX3 KO, PRDX5 KO, and PRDX3/5 DKO cells. Both cytosol- and (surprisingly) mitochondrial matrix-targeted HyPer7 showed only minor changes compared to WT cells (Fig. 1C). In the cytosol, deletion of mitochondrial matrix PRDXs led to a slight decrease in the HyPer7 steady state, whereas in the mitochondrial matrix, PRDX3 KO and PRDX3/5 DKO cells exhibited a modest but significant increase in the HyPer7 BL (Fig. 1C, *zoom-in*). This contrasts with loss of cytosolic PRDXs: while WT, PRDX1 KO and PRDX2 KO cells exhibited very similar HyPer7 BL, PRDX1/2 DKO cells had a markedly more oxidized HyPer7 BL in both compartments highlighting the importance of cytosolic antioxidant systems for maintaining matrix redox homeostasis (Fig. 1D).

Next, we examined the dynamic behaviour of the HyPer7 sensor following the bolus application of exogenous H_2_O_2_. HyPer7 is significantly more sensitive than previous H_2_O_2_ sensors and responds to exogenous bolus H_2_O_2_ applications at concentrations as low as 2 μM [22]. When we applied 5 μM external H_2_O_2_, WT cells exhibited a substantial oxidative deflection in cytosolic HyPer7 that after reaching a peak, 15 min after bolus H_2_O_2_ application, slowly returned towards basal HyPer7 oxidation levels (Fig. 1E, *left graph*). This response was attenuated in PRDX3 and PRDX5 KO cells, suggesting a possible adaptation of cytosolic antioxidative capacity upon challenges in mitochondrial redox homeostasis, similar to previous observations in baker's yeast and human cells [22,31]. In the mitochondrial matrix, as expected for a compartment shielded from exogenous influences by the cytosol, the HyPer7 deflection upon 5 μM H_2_O_2_ was minimal and difficult to analyse (Fig. 1E, *middle graph*). To improve resolution, we applied 10 μM H_2_O_2_, revealing that PRDX3 deletion sensitized the HyPer7 response, whereas PRDX5 deletion again blunted it (Fig. 1E, *right graph*). This aligns with activity data in human cells, which indicate that PRDX3 has an activity level (concentration x rate constant of oxidation) that is 50–500 times higher than PRDX5 [32]. In PRDX3/5 DKO cells, the HyPer7 response was more pronounced in both compartments (Fig. 1F).

By contrast, PRDX1 KO cells exhibited a strong HyPer7 response to 5 μM H_2_O_2_ in both compartments (Fig. 1G), which became even more pronounced in PRDX1/2 DKO cells (Fig. 1H). When comparing the AUC for PRDX1 KO, PRDX3 KO, and PRDX3/5 DKO, we found that PRDX1 deletion led to a four-fold stronger HyPer7 response in the mitochondrial matrix (Fig. 1I).

Collectively, these findings indicate that cytosolic PRDX1 is not only the primary regulator of cytosolic but also of mitochondrial matrix H_2_O_2_ levels and responses following exogenous H_2_O_2_ application.

### 2.2 Cells chemo-genetically generating matrix H_2_O_2_ rely on PRDX1 to control mitochondrial matrix H_2_O_2_ levels and dynamics

**Fig. 2:**
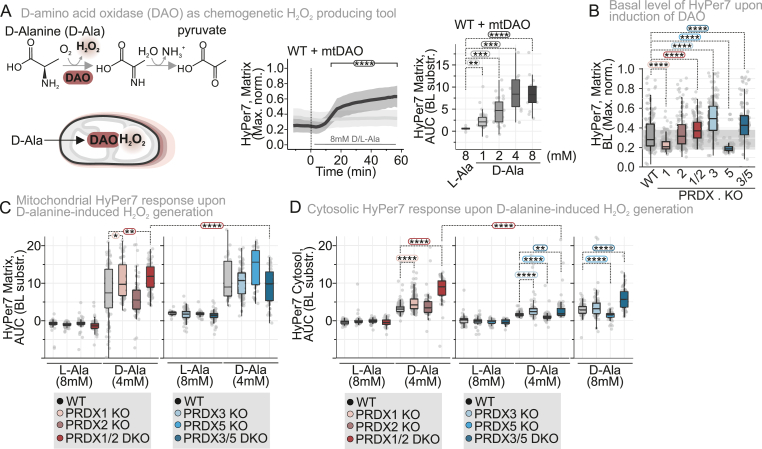
**PRDX1 is the main determinant of mitochondrial matrix H_2_O_2_ levels and dynamics in cells chemogenetically generating mitochondrial H_2_O_2_** (A) Mechanism of d-amino acid oxidase (DAO) in the production of H_2_O_2_. Cell lines stably expressing a mitochondria-targeted DAO (mtDAO) were generated and tested. The H_2_O_2_ production of the wild type cell line with mtDAO was assessed with the mitochondrial localized HyPer7. AUC of the HyPer7 oxidation upon increasing concentrations of d-Alanine was calculated for the time period of 20 to 55 min. Cells were grown in glucose-containing medium. The number of cells, number of experimental replicates and information on statistical analysis and visualization can be found in **Supplementary dataset S1**. (B) Basal level (BL) of mitochondrial targeted HyPer7 upon induction of the mtDAO expression. Cells were grown in glucose-containing medium. The number of cells, number of experimental replicates and information on statistical analysis and visualization can be found in **Supplementary dataset S1**. (C) AUC of the mitochondria-targeted HyPer7 oxidation upon treatment with 4 mM d-Alanine or 8 mM L-Alanine. AUC was calculated for the time period of 20 to 55 min. Cells were grown in glucose-containing medium. The number of cells, number of experimental replicates and information on statistical analysis and visualization can be found in **Supplementary dataset S1**. (D) AUC of the cytosolic targeted HyPer7 oxidation upon treatment with 4 mM or 8 mM d-Alanine or 8 mM L-Alanine. The AUC was calculated for the time period of 20 to 55 min. Cells were grown in glucose-containing medium. The number of cells, number of experimental replicates and information on statistical analysis and visualization can be found in **Supplementary dataset S1**. ∗P ≤ 0.05, ∗∗P ≤ 0.01, ∗∗∗P ≤ 0.001, ∗∗∗∗P ≤ 0.0001.

To exclude the influence of cytosolic antioxidative systems on modulating the flux of exogenously applied H_2_O_2_ *en route* to mitochondria, we turned to a genetically engineered system capable of titratable H_2_O_2_ production within the mitochondrial matrix. To this end, we generated stable, inducible cell lines expressing a mitochondrial matrix‐targeted, C‐terminally FLAG‐tagged d‐amino acid oxidase (mtDAO) (Fig. 2A and S2; [14,33]). In the presence of D-Ala, but not L-Ala, mtDAO produces H_2_O_2_ in a dose‐dependent manner within the mitochondrial matrix reaching a plateau after approximately 40-45 min (Fig. 2A). The use of stable cell lines ensures homogeneous mtDAO expression across all cells, while inducible expression minimizes potential adaptation effects associated with continuous oxidase activity. Despite these precautions, we observed a slight increase in HyPer7 BL in mtDAO-expressing cells, even in the absence D-Ala addition (Fig. 2B, *compare to* Fig. 1C and D), indicating continuous mitochondrial H_2_O_2_ generation. It mainly affected PRDX3 KO and PRDX3/5 DKO cells emphasizing the importance of PRDX3 as scavenger of H_2_O_2_ in in the mitochondrial matrix. Curiously, both PRDX2 KO and PRDX1/2 DKO cells also exhibited elevated mitochondrial matrix HyPer7 BL, indicating that H_2_O_2_ scavenging by cytosolic PRDX might provide a protective mechanism for mitochondrial matrix generated H_2_O_2_.

We then used this system to monitor HyPer7 responses in different PRDX KO cells. In all cell lines, addition of 4 mM D-Ala but not L-Ala resulted in an increased HyPer7 AUC (Fig. 2C). In PRDX1 KO and in particular in PRDX1/2 DKO cells, the mitochondrial matrix HyPer7 oxidation was more pronounced compared to WT cells (Fig. 2C). This also held true at lower D-Ala concentrations of 1 mM (Fig. S2B). Conversely, KO of the mitochondrial matrix PRDXs did not result in a more increased HyPer7 AUC in the mitochondrial matrix compared to the WT control.

In the cytosol, a substantial HyPer7 response to mtDAO-dependent H_2_O_2_ generation was observed in PRDX1 KO and PRDX1/2 DKO cells (Fig. 2D–S2C) in line with the protective role of PRDX1 for cytosolic redox homeostasis. PRDX3 KO and PRDX3/5 DKO cells also exhibited elevated response for cells expressing cytosolic HyPer7 fitting to the role of PRDX3 as more prominent H_2_O_2_ scavenger compared to PRDX5 (Fig. 2D–S2C). A direct comparison of PRDX1/2 DKO and PRDX3/5 DKO cells showed that the former exhibited a stronger deflection of matrix HyPer7 even in the case of this engineered mitochondrial matrix–H_2_O_2_–generating system (Fig. S2D).

This suggests that the cytosol and its 2-Cys PRDXs serves as a significant sink for mitochondrial H_2_O_2_. Collectively, these mtDAO-based experiments underscore the dominant role of PRDX1 in regulating mitochondrial H_2_O_2_ levels and fluxes for both exogenous and mitochondria-derived H_2_O_2_.

### 2.3 Modelling indicates high IMM permeability and supports a role for the cytosol as sink for mitochondrial H_2_O_2_

The relative influence of the cytosolic *versus* the matrix PRDXs against H_2_O_2_ generated in the matrix depends on a complex interplay of factors. Namely, the relative concentrations of these proteins, the activity of alternative H_2_O_2_ sinks, the extent to which the H_2_O_2_ generation rate overloads the compartment's reductive capacity, the permeability of the mitochondrial membranes, *etc*. To gain further insights into the factors that allow PRDX1 to so strongly influence matrix H_2_O_2_, we resorted to kinetic modelling. Previous kinetic modelling of H_2_O_2_ dynamics in the mitochondria of HeLa cells [32] highlighted - in line with experimental findings ([23], Fig. 1E) - PRDX3 as the major determinant in controlling mitochondrial H_2_O_2_ dynamics. However, this was only true when H_2_O_2_ was locally generated with nM s^−1^ to low μM s^−1^ fluxes [32]. Other matrix antioxidant enzymes such as GPX1, GPX4 and PRDX5 did not play a relevant role in controlling matrix H_2_O_2_. This study already indicated that efflux from the matrix was important for the H_2_O_2_ dynamics in this compartment; however, it overlooked the role of cytosolic H_2_O_2_ metabolism.

**Fig. 3:**
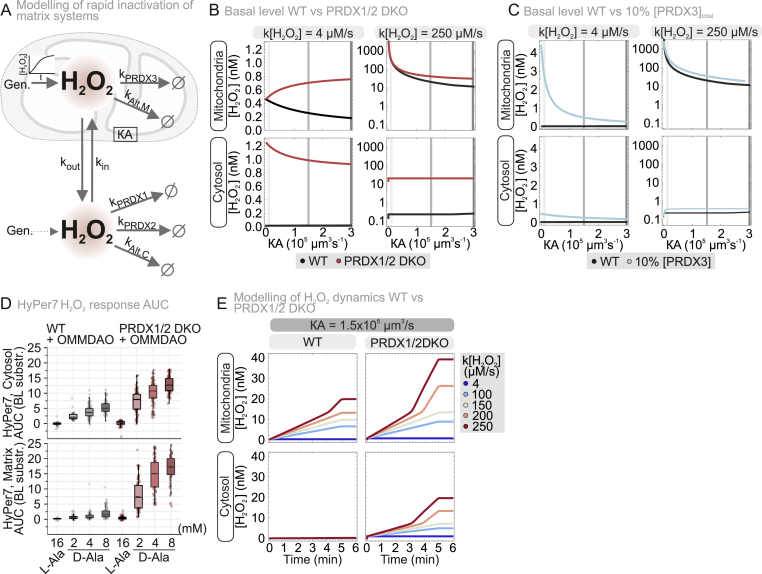
**Limited reductive regeneration of PRDX3 renders the cytosol the sink for mitochondrial H_2_O_2_.** (A) Simplified model utilized for modeling of the mitochondrial H_2_O_2_ concentrations and dynamics in HEK293 cells. (B) Cellular basal H_2_O_2_ levels upon loss of PRDX1 and PRDX2, upon increased mitochondrial H_2_O_2_ production (k[H_2_O_2_] 4 and 250 μM s^−1^) with increased H_2_O_2_ release (*КA* = 0.01 – 3.0 × 10^5^ μm^3^ s^−1^) (C) Cellular basal H_2_O_2_ levels upon 10% [PRDX3], upon increased mitochondrial H_2_O_2_ production (k[H_2_O_2_] 4 and 250 μM s^−1^) with increased H_2_O_2_ release (*КA* = 0.01 – 3.0 × 10^5^ μm^3^ s^−1^). (D) Mechanism of d-amino acid oxidase (DAO) in the production of H_2_O_2_. Cell lines stably expressing a mitochondria-targeted DAO (mtDAO) were generated and tested. The H_2_O_2_production of the wild type cell line with mtDAO was assessed with the mitochondrial localized HyPer7. AUC of the HyPer7 oxidation upon increasing concentrations of d-Alanine was calculated for the time period of 20 to 55 min. Cells were grown in glucose-containing medium. The number of cells, number of experimental replicates and information on statistical analysis and visualization can be found in **Supplementary dataset S1**. (E) Cellular H_2_O_2_ dynamics upon loss of PRDX1 and PRDX2, upon increased mitochondrial H_2_O_2_ production (k[H_2_O_2_] 4 and 250 μM s^−1^) with H_2_O_2_ release (*КA* = 1.5 × 10^5^ μm^3^ s^−1^).

We thus adapted the previous model to better simulate mitochondria in intact HEK293 cells and extended it by coupling it with a model of the cytosolic H_2_O_2_ metabolism (Supplementary methods S1 and 2). In the new model, matrix H_2_O_2_ is removed by PRDX3 and several minor alternative pathways (collectively denoted by “Alt,M″ in Fig. 3A), and exchanges with the cytosol (“out”/“in”, Fig. 3A). H_2_O_2_ becomes released across the mitochondrial membranes, for which we assume varying *permeability surface values КA*, *i.e.* products of membrane permeability *К* and surface area *A* for the IMM (Supplementary method S3). In the cytosol, H_2_O_2_ is handled by PRDX1 (as major antioxidant), by PRDX2, and by a sum of minor alternative H_2_O_2_ removal pathways (“Alt,C” in Fig. 3A). Concentrations and rate constants of involved antioxidative systems were derived from the literature and in some cases concentration ranges were assumed and modelled (Table S7).

We first modelled PRDX3 redox dynamics under increasing mitochondrial H_2_O_2_ flux and found that while low production rates (≤100 μM s^−1^, which corresponds to ca 2 mM D-Ala when using the mtDAO system) maintain PRDX3 in a reduced, active state, higher fluxes rapidly inactivate the system via TRX2 oxidation (Fig. S3A and B). Incorporating mitochondrial H_2_O_2_ release and cytosolic scavenging revealed that sufficient membrane permeability (*КA* ≥ 1.5 × 10^5^ μm^3^ s^−1^) preserves PRDX3/TRX2 in a reduced state even at elevated H_2_O_2_ generation (Fig. S3A and B), indicating a critical role of H_2_O_2_ efflux and cytosolic buffering. Analysis of matrix H_2_O_2_ dynamics showed that without mitochondrial H_2_O_2_ release (*КA* = 0), matrix H_2_O_2_ accumulates indefinitely, and even low H_2_O_2_ permeability of the IMM (*КA* = 0.01 × 10^5^ μm^3^ s^−1^) still yields unrealistically high μM levels compared to measured concentrations (Fig. S3C–E). Only assuming higher IMM permeability reduced matrix H_2_O_2_ to the nM range, suggesting an IMM permeability at least an order of magnitude greater than the plasma membrane (PM, Fig. S3C–E, further supported by a sensitivity analysis - Supplementary method S4). Such a high IMM permeability is in line with previous measurements on isolated rat liver mitochondria that indicated 50-fold higher IMM permeabilities compared to the PM [34].

We next assessed the effects of PRDX1/2 loss and PRDX3 depletion. Under basal conditions (continuous generation of 4 μM s^−1^ and 0.2 μM s^−1^ in matrix and cytosol, respectively), we found low nanomolar H_2_O_2_ levels in matrix and cytosol that were sensitive to PRDX1/2 loss depending on *КA* (Fig. 3B and C). The WT steady state values thereby are in accordance with the literature [1,20,32]. At high matrix H_2_O_2_ generation (250 μM s^−1^), absence of cytosolic PRDXs increased H_2_O_2_ in both compartments - especially at higher *КA* - supporting the cytosol as a sink, while PRDX3 depletion mainly affected matrix H_2_O_2_ at low *КA* (Fig. 3B and C). Since PRDX1/2 loss had the strongest impact at higher *КA* and exceeded that of PRDX3 depletion, these results again support high IMM permeability.

To test mitochondrial membrane permeability experimentally in intact cells, we targeted DAO to the cytosolic side of the OMM and monitored HyPer7 oxidation in cytosol and matrix (Fig. 3D–S3F,G). This setup bypasses PM and cytosolic diffusion, revealing stronger HyPer7 responses in PRDX1/2 DKO cells and increased matrix signals upon OMM-derived H_2_O_2_. These results show that PRDX1/2 limit H_2_O_2_ spread from the OMM, while detection of OMM-generated H_2_O_2_ in the matrix - even in WT cells - supports very high permeability of both mitochondrial membranes.

Lastly, we modelled H_2_O_2_ dynamics in WT vs PRDX1/2 DKO cells, and found at all H_2_O_2_ generation rates in particular at 200 and 250 μM s^−1^ a strong impact of PRDX1/2 loss on matrix H_2_O_2_ levels that became more pronounced upon higher *КA* (Fig. 3E–S3H).

Collectively, our modelling approach supports that mitochondrial antioxidant systems can maintain H_2_O_2_ homeostasis at basal H_2_O_2_ flux conditions reasonably well. However, under conditions of increased flux as it may occur upon *e.g.* fluctuating oxygen tensions or insufficient electron transport through the respiratory chain, cytosolic PRDXs become critical contributors to maintain low matrix H_2_O_2_ levels by establishing strong gradients across the mitochondrial membranes. Notably, our modelling supports that this is only possible if the IMM has a very high permeability for H_2_O_2_.

### 2.4 PRDX1 loss sensitises cells to exogenous bolus H_2_O_2_- and mitochondrial H_2_O_2_ -induced cell death

**Fig. 4:**
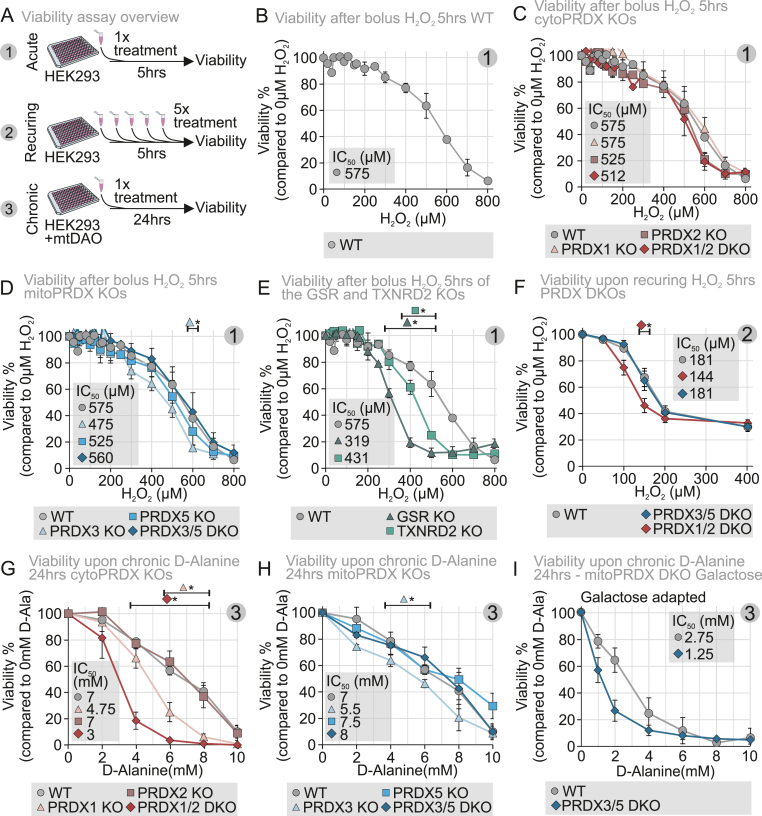
**Viability following exposure to mitochondrial-generated H_2_O_2_ shows a significant sensitization of cells upon PRDX1 loss.** (A) Assessing cellular viability by utilizing the cellular capacities to convert resazurin to resorufin. Three measurement setups were utilized: (1) acute, in which cells were treated with a bolus of H_2_O_2_ for 5 h; (2) recurring, in which cells were treated with a bolus of H_2_O_2_ every hour for 5 h; (3) chronic, in which the mtDAO system was utilized to generate chronic 24 h H_2_O_2_ stress by the addition of d-Alanine. (B) Viability of the wild type cell line was dose-dependently affected upon increasing concentrations of H_2_O_2_ when treated for 5 h. IC_50_ was calculated to be around 575 μM. The cells were grown in glucose-containing medium. Symbols represents average over three experimental replicates each with eight technical replicates, and the standard deviation is represented by the whiskers. Significance was assessed using the Welch's *t*-test. (C) Viability of the cytosolic PRDX KO cell lines did not differ from the wild type cell line when treated with acute H_2_O_2_ stress (grey/circle, wild type; pink/triangle, PRDX1 KO; brown/square PRDX2 KO; dark red/diamond, PRDX1/2 DKO). IC_50_ was calculated to be around 575 μM for the wild type and PRDX1 KO, 525 μM for the PRDX2 KO and 512 μM for the PRDX1/2 DKO cell line. The cells were grown in glucose-containing medium. Symbols represents average over three experimental replicates each with eight technical replicates, and the standard deviation is represented by the whiskers. Significance was assessed using the Welch's *t*-test. (D) Viability of the mitochondrial PRDX KO cell lines did not differ from the wild type cell line when treated with acute H_2_O_2_ stress (grey/circle, wild type; light blue/triangle, PRDX3 KO; blue/square PRDX5 KO; dark blue/diamond, PRDX3/5 DKO). IC_50_ was calculated to be around 575 μM for the wild type, 475 μM for the PRDX3 KO, 525 μM for the PRDX5 KO and 560 μM for the PRDX3/5 DKO cell line. The cells were grown in glucose-containing medium. Symbols represents average over three experimental replicates each with eight technical replicates, and the standard deviation is represented by the whiskers. Significance was assessed using the Welch's *t*-test. (E) Viability of GSR KO and TXNRD2 KO cell lines sensitized cells to acute H_2_O_2_ stress, when compared to the wild type cell line (grey/circle, wild type; dark green/triangle, GSR KO; light green/square TXNRD2 KO). IC_50_ was calculated to be around 575 μM for the wild type, 319 μM for the GSR KO and 431 μM for the TXNRD2 KO cell line. The cells were grown in glucose-containing medium. Symbols represent average over three experimental replicates each with eight technical replicates, and the standard deviation is represented by the whiskers. Significance was assessed using the Welch's *t*-test. (F) Viability of the PRDX DKO cell lines sensitized the cells to recurring H_2_O_2_ stress, compared to the wild type cell line (grey/circle, wild type; dark red/diamond, PRDX1/2 DKO; dark blue/diamond, PRDX3/5 DKO). IC_50_ was calculated to be around 181 μM for the wild type, 144 μM for the PRDX1/2 DKO and 181 μM for the PRDX3/5 DKO cell line. The cells were grown in glucose-containing medium. Symbols represent average over three experimental replicates each with eight technical replicates, and the standard deviation is represented by the whiskers. Significance was assessed using the Welch's *t*-test. (G) Viability of the cytosolic PRDX KO cell lines was sensitized upon chronic H_2_O_2_ stress, compared to the wild type cell line (grey/circle, wild type; pink/triangle, PRDX1 KO; brown/square PRDX2 KO; dark red/diamond, PRDX1/2 DKO). IC_50_ was calculated to be around 7 mM for the wild type and PRDX2 KO, 4.75 mM for the PRDX1 KO and 3 mM for the PRDX1/2 DKO cell line. The cells were grown in glucose-containing medium. Symbols represent average over three experimental replicates each with eight technical replicates, and the standard deviation is represented by the whiskers. Significance was assessed using the Welch's *t*-test. (H) Viability of the mitochondrial PRDX KO cell lines did not differ from the wild type cell line when treated with chronic H_2_O_2_ stress (grey/circle, wild type; light blue/triangle, PRDX3 KO; blue/square PRDX5 KO; dark blue/diamond, PRDX3/5 DKO). IC_50_ was calculated to be around 7 mM for the wild type, 5.5 mM for the PRDX3 KO, 7.5 mM for the PRDX5 KO and 8 mM for the PRDX3/5 DKO cell line. The cells were grown in glucose-containing medium. Symbols represent average over three experimental replicates each with eight technical replicates, and the standard deviation is represented by the whiskers. Significance was assessed using the Welch's *t*-test. (I) Galactose grown mitochondrial PRDX DKO cell line were more sensitive upon chronic H_2_O_2_ stress compared to the wild type cell line (grey/circle, wild type; dark blue/diamond, PRDX3/5 DKO). IC_50_ was calculated to be around 2.75 mM for the wild type and 1.25 mM for the PRDX3/5 DKO cell line. The cells were grown in galactose-containing medium for minimum 2 weeks. Symbols represent average over three experimental replicates each with eight technical replicates, and the standard deviation is represented by the whiskers. Significance was assessed using the Welch's *t*-test. ∗P ≤ 0.05, ∗∗P ≤ 0.01, ∗∗∗P ≤ 0.001, ∗∗∗∗P ≤ 0.0001.

Our direct measurements and modelling of H_2_O_2_ levels and dynamics in the cytosol and mitochondrial matrix suggest that cytosolic PRDXs play a dominant role not only in managing cytosolic H_2_O_2_ but also in regulating mitochondrial H_2_O_2_. To further investigate the physiological consequences of PRDX loss, we examined cell viability under acute, recurring and chronic H_2_O_2_ stress conditions (Fig. 4A).

First, we subjected WT cells grown in glucose-containing medium to an acute bolus of exogenous H_2_O_2_ at varying concentrations (Fig. 4B). After 5 h, we assessed cell viability and observed a dose-dependent decrease, with an IC_50_ of approximately 575 μM H_2_O_2_. Notably, all PRDX KO and DKO cell lines exhibited similar responses in this assay with the exception of the PRDX3 KO which seemed to be slightly more sensitive (Fig. 4C and D), indicating that PRDXs are not essential for survival under this acute H_2_O_2_ shock regime. In contrast, deletion of two other antioxidant enzymes—glutathione reductase (GSR) and mitochondrial thioredoxin reductase 2 (TXNRD2)—had a significant impact, as both KO cell lines showed markedly lower IC_50_ values of 319 μM and 431 μM, respectively (Fig. 4E). We then repeated the experiment with recurring bolus H_2_O_2_ application (5x application, every hour for 5 h). While PRDX3/5 DKO cells had an IC_50_ very similar to WT cells, PRDX1/2 DKO cells appeared to be more sensitive to this stress (Fig. 4F)

To further assess the effects of chronic mitochondrial oxidative stress, we utilized mtDAO-expressing cell lines grown in glucose-containing medium. MtDAO-expressing WT cells were treated with D-Ala or, as a control, L-Ala for 24 h before cell viability was measured. While L-Ala had no effect (Fig. S4A), D-Ala induced a gradual decrease in viability with increasing concentrations, yielding an IC_50_ of approximately 7 mM (Fig. 4G). The PRDX KO cell lines exhibited varying responses: PRDX5 KO, PRDX2 KO, and PRDX3/5 DKO cells displayed IC_50_ values similar to WT (7,5 mM, 7 mM, and 8 mM, respectively) (Fig. 4G and H). However, PRDX3 KO, PRDX1 KO, PRDX1/2 DKO cells showed significantly reduced IC_50_ values of 5.5 mM, 4.75 mM and 3 mM, respectively (Fig. 4G and H). Lastly, we tested whether PRDX3/5 DKO cells would be more susceptible to chronic mitochondrial stress treatment upon growth in galactose-containing medium, a condition that requires respiratory chain use. We thereby found a clearly reduced IC_50_ for PRDX3/5 DKO cells compared to the WT (Fig. 4I). Increased sensitivity to cell death in our PRDX1/2 DKO cells was likely not due to ferroptosis induction as the GPX4-inhibitor RSL3 did not trigger additional cell death (Fig. S4B). This was different for the PRDX6 KO cells that served as positive controls and are known to be susceptible to ferroptosis [[35], [36], [37]].

Together, these findings further underscore the critical role of cytosolic PRDXs in particular PRDX1 in counteracting mitochondrial H_2_O_2_ stress and maintaining mitochondrial H_2_O_2_ homeostasis. This made us wonder which factors, in particular mitochondria-linked ones, might compensate in case of the loss of this cytosolic protective antioxidative peroxiredoxin machinery.

### 2.5 A CRISPR screen identifies genetic effectors of cellular sensitivity to mitochondria-generated chronic H_2_O_2_ stress

**Fig. 5:**
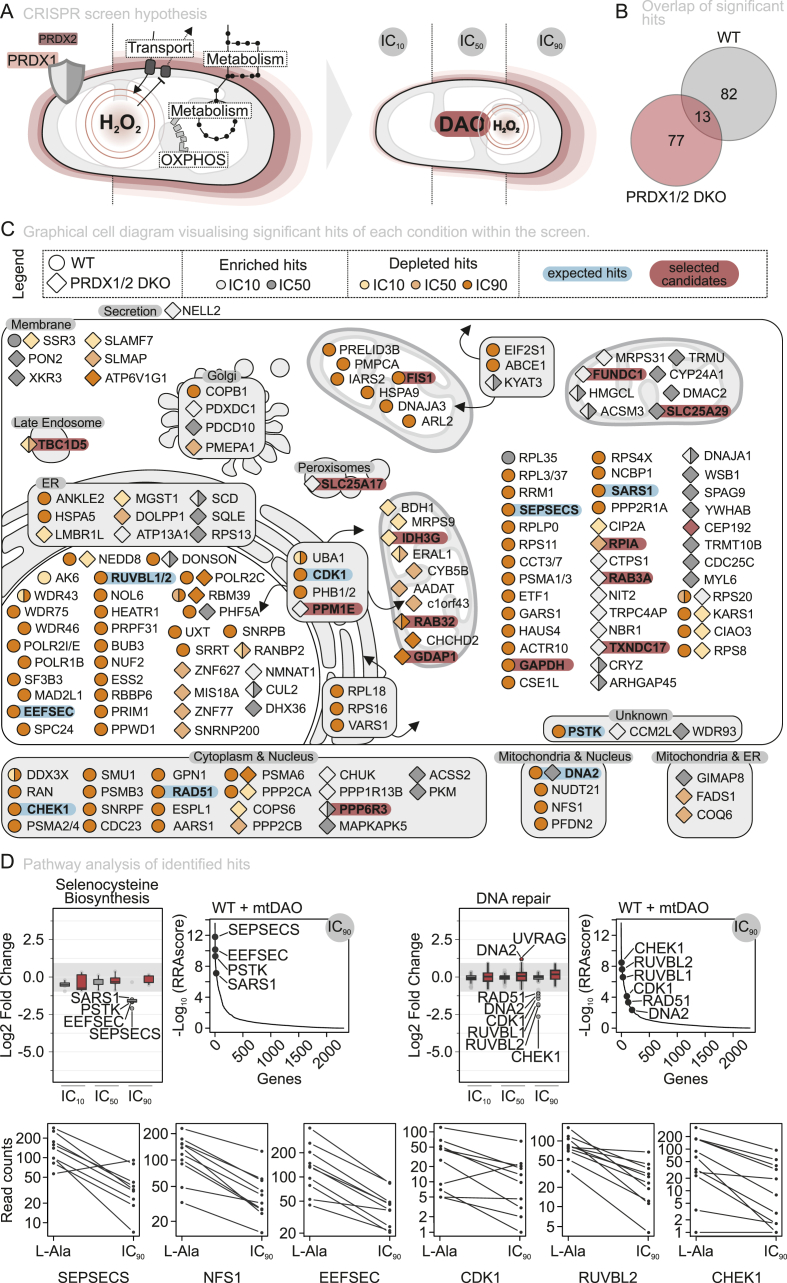
A mitochondrion-focussed CRISPR screen reveals players in handling mitochondrion-generated H_2_O_2_. (A) Graphical overview of the working hypothesis (left) and schematic representation of the CRISPR screen design (right), illustrating the three experimental conditions used in the screen. (B) Overview of significant hits (LFC -1 < or >1, and p < 0.05) across the three conditions compared to the l-Alanine control of each specific cell line. (C) Graphical cellular overview of significant hits identified under each condition. Wild type (WT) cells are shown as circles and PRDX1/2 DKO cells as diamonds. Protective hits (associated with increased cellular viability) are shown in grey, whereas sensitizing hits (associated with decreased viability) are shown in orange. Expected hits are highlighted in blue, and selected candidate hits are highlighted in red. (D) Functional characterization via GO term analysis, involving DNA repair and selenocysteine biosynthesis. Visualized via boxplot LFC (top), in which the boxplot visualizes the median, 1st and 3rd quartiles, and the whiskers 1.5 × IQR. Colored and mentioned circles are hits which were significant (LFC -1 < or >1 and p ≤ 0.05). The RRA score (top) and sgRNA count (bottom) of hits that prove the functionality of the screen in the wild-type cell line expressing the mtDAO.

To systematically identify genetic factors that modulate cellular sensitivity to mitochondria-derived oxidative stress, we designed a CRISPR/Cas9 single guide RNA (sgRNA) library focused on mitochondrial proteins (MitoCarta) and their closest co-dependent proteins (DEPmap) and conducted drop-out screens in WT and PRDX1/2 DKO cells. During the screen, these cells continuously generated H_2_O_2_ via mtDAO induction and D-Ala addition. We performed the screen in glucose-containing medium to avoid challenges arising from deletions of genes important for respiratory growth (Fig. 5A, S5A).

For the sgRNA library design, we preselected 3050 unique genes (*i.e.* mitochondrial genes from GO, MitoCarta and IMPI and their top co-dependent genes from DepMap, in addition to aquaporins, which were previously linked to mitochondrial H_2_O_2_ transport). Further refinement led to the inclusion of 2308 unique genes that corresponded to 21,068 unique gRNAs of the CRISPR knockout library (Fig. S5B). We then infected cells with the sgRNA library, followed by selection with puromycin. The infected cells were then passaged and either left untreated or treated with D-Ala or L-Ala after mtDAO induction. D-Ala was administered at IC_10_, IC_50_, and IC_90_ concentrations in both WT and PRDX1/2 DKO cells (Fig. S5C). The frequencies of sgRNA elements in each cell population were determined by next-generation sequencing and analysed using MAGeCK [38]. Under these conditions, we expect that sgRNAs targeting genes whose corresponding proteins serve in the defense against H_2_O_2_ would likely become depleted (“sensitizing hits“). Conversely, a sgRNA targeting a gene whose gene product would normally impair the handling of oxidative stress would become enriched (“protective hits“). We identified a total of 172 gene hits (false discovery rate (FDR) < 0.5, P-value <0.05, log_2_ fold-change (LFC) > 0.5) from the CRISPR/Cas9 screen (Fig. 5B,C, S5D, Supplementary Dataset 3).

When analyzing the distribution of these hits across the different conditions, we observed in WT cells an increasing number of hits with increasing D-Ala treatment concentrations up to IC_90_ (Fig. S5E). Conversely, with PRDX1/2 DKO cells, we observed most hits at IC_10_ and IC_50_. Notably, only in the PRDX1/2 DKO we did find a prominent enrichment of sgRNAs. MitoCarta3.0 cross-referencing and gene ontology (GO) term analysis revealed no particular enrichment of specific key processes which is not surprising as we worked with a targeted sgRNA library (Fig. S5F and G). The exceptions were selenoprotein biosynthesis and DNA repair, as sgRNAs targeting genes adhering to these GO terms were depleted in particular at IC_90_. For selenoprotein biosynthesis we found a significant depletion of SARS1, PSTK, EEFSEC, and SEPSECS in particular at IC_90_ (Fig. 5D). For DNA repair, we observed a depletion of RAD51, DNA2, CDK1, RUVBL1 and 2, and CHECK1 (Fig. 5D). Selenoproteins like thioredoxin reductases and glutathione peroxidases take critical roles in antioxidative defense, and DNA repair is critical to correct errors induced by oxidative stress. The depletion of the sgRNAs targeting the respective genes thus underscores the importance of the encoded proteins to counteract oxidative stress originating from mitochondria.

We further identified sgRNAs significantly depleted that target genes encoding for proteins involved in cellular NADPH regeneration (Fig. 5C): these included GAPDH (important for NADH generation in glycolysis and as switch to activate the pentose phosphate pathway), RPIA (ribose 5-phosphate isomerase, an enzyme of the pentose phosphate pathway), and IDH3G (contributes to NADPH regeneration in the matrix).

We also found some sgRNAs significantly enriched that target genes encoding for proteins involved in further redox processes (Fig. 5C). These included TXNDC17 (gene encoding for TRP14, the rate-limiting enzyme for intracellular cystine reduction which is required for glutathione synthesis), and SLC25A17 (important for cofactor delivery to peroxisomes). The enrichment of these sgRNAs might indicate a functional rerouting, *e.g.* to prevent TRP14 from utilizing too much NADPH or prevent import of cofactors into peroxisomes that are required elsewhere.

Additionally, sgRNA enrichment was observed for SLC25A29, which transports basic amino acids into mitochondria as well as PPM1E and PPP6R3, two genes for which silencing has been linked to increased AMPK activation (Fig. 5C). SLC25A29 has been reported to increase mitochondria-derived nitric oxide due to its role in arginine transport. PRDX5 was implied in handling peroxynitrite, the product of the reaction of nitric oxide with superoxide anions. Loss of SLC25A29 might thus supress peroxynitrite generation and thus be protective for mitochondria under increased ROS load.

Lastly, we also observed changes in sgRNAs targeting different players in vesicular and membrane transport (*e.g.* TBC1D5, RAB3A, RAB32, GDAP1), in the maintenance of mitochondrial contact sites and mitochondrial dynamics (*e.g.* FIS1, FUNDC1), and in proteostasis regulation (subunits of the mitochondrial and cytosolic ribosomes, proteasome subunits) (Fig. 5C). The modulation of the proteostasis landscape and of membrane dynamics are important hallmarks of stress responses.

Together, our results provide an exciting view of the diverse mechanisms governing cellular resilience to chronic mitochondrial oxidative stress.

### 2.6 Mitophagy acts as a compensatory mechanism to ensure cell survival in the absence of PRDX1-dependent mitochondrial redox protection

**Fig. 6:**
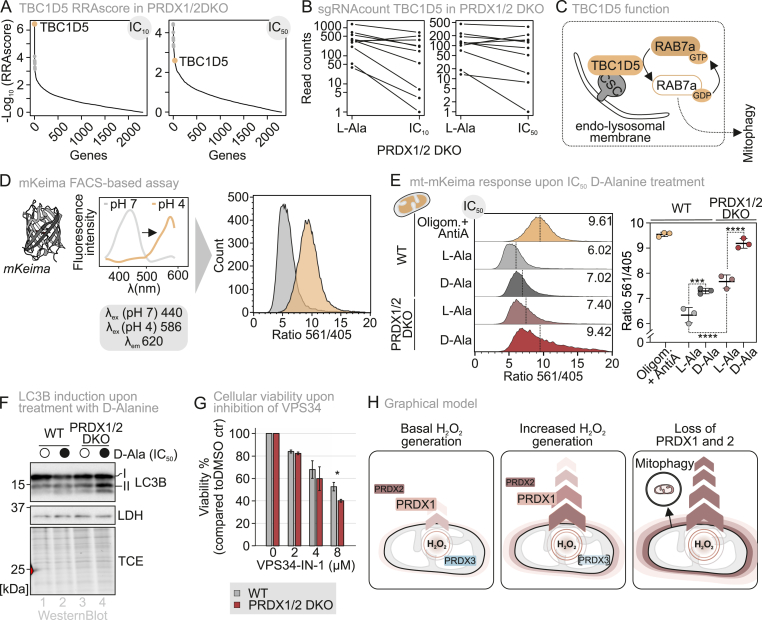
Increased mitophagy compensates for the loss of PRDX1. (A) RRA score of the PRDX1/2 DKO cell line expressing the mtDAO upon IC_10_ and IC_50_d-Alanine treatment. (B) sgRNA count of each measured sgRNA of TBC1D5 in the PRDX1/2 DKO cell line expressing the mtDAO upon IC_10_ and IC_50_d-Alanine treatment. (C) Graphical illustration of TBC1D5 functions. (D) Mt-mKeima and mKeima- FIS1^MTS^ measurement setup and representation of data. A ratiometric signal is obtained and visualized via a histogram. (E) FACS results of steady state mt-mKeima in wild type (WT, grey) and PRDX1/2 DKO (dark red) cell lines expressing the mtDAO system, when treated with D/l-Alanine for 8 h. The histogram (*left*) is a representative FACS result of three experimental replicates. The scatter plot (*right*) represents these three experimental replicates, visualising the mean and standard deviation. Significance was assessed using the two-way ANOVA test. (F) Immunoblot analysis of WT and PRDX1/2 double knockout cells treated for 8 h with IC_50_ D/l-Alanine. (G) Viability of the PRDX1/2 DKO cell line was sensitized upon inhibition of VPS34, compared to the wild type cell line (grey, WT; dark red, PRDX1/2 DKO). The cells were grown in glucose-containing medium and treated for 20 h. The bar diagram represents the average over three experimental replicates, each with eight technical replicates, and the standard deviation is represented by the whiskers. Significance was assessed using the Welch's *t*-test. (H) Graphical overview. Cytosolic antioxidant systems can act as H_2_O_2_ sinks to maintain matrix H_2_O_2_ homeostasis. ∗P ≤ 0.05, ∗∗P ≤ 0.01, ∗∗∗P ≤ 0.001, ∗∗∗∗P ≤ 0.0001.

Among the highest scoring hits was TBC1D5, whose deletion sensitized in particular PRDX1/2 DKO cells to mtDAO-generated oxidative stress. The RRA score yielded it as the top hit at the IC_10_ and one of the top hits at the IC_50_ D-Ala concentrations (Fig. 6A). Analyzing the effects of the ten individual sgRNAs targeting the TBC1D5 gene revealed that these were consistently depleted from the pool at chronic stress conditions (Fig. 6B).

TBC1D5 encodes a GTPase-activating protein (GAP) for Rab7a, a key regulator of mitophagy [[39], [40], [41]] (Fig. 6C). Given that mitophagy selectively removes damaged mitochondria and that previous studies suggest oxidative stress can induce mitophagy [[42], [43], [44]], we further investigated whether PRDX1/2 loss increased mitophagic flux. To this end, we employed a FACS-based mKeima assay (Fig. 6D, [[45], [46], [47]]). This fluorescent protein is pH-sensitive, and acid-stable and has two excitation peaks with maxima at 440 nm in neutral environments and 586 nm in acidic environments. From this bimodal excitation, a ratiometric signal can be calculated to quantify mitophagy in live cells. By targeting this probe to the mitochondrial matrix, already without D-Ala treatment, PRDX1/2 DKO cells expressing mtDAO exhibited an increased mitophagic flux compared to WT cells (Fig. 6E). D-Ala treatment increased mitophagy significantly in both PRDX1/2 DKO and WT; for the PRDX1/2 DKO to an extend that it matches treatment with oligomycin and antimycin A used as positive control for mitophagy induction (Fig. 6E). We confirmed these data with the OMM-targeted mKeima-FIS1^MTS^ (Fig. S6).

We complemented the mKeima experiment by assessing the lipidation state of microtubule-associated protein light chain 3 (LC3). We thereby found that the lipidated autophagosome-bound LC3-II form was prominently appearing in PRDX1/2 DKO cells upon induction of mitochondrial H_2_O_2_ production (Fig. 6F).

If mitophagy represents a backup strategy for the lack of efficient cytosolic H_2_O_2_ degradation, inhibition of the process should affect PRDX1/2 DKO cells more than WT. We thus employed the inhibitor VPS34-IN-1, which targets VPS34 thereby inhibiting autophagy and vesicle trafficking. We found that incubation of cells with 8 μM VPS34-IN-1 in the absence of H_2_O_2_ significantly decreased viability of PRDX1/2 DKO cells compared to WT (Fig. 6G).

In conclusion, we demonstrate that mitophagy is increased as a consequence of sustained mitochondrial oxidative stress, and we propose that it serves as a compensatory mechanism for the loss of PRDX-mediated cytosolic protection of mitochondria (Fig. 6H).

## 3 Discussion

### 3.1 Cytosolic PRDX1 acts as a major scavenger for mitochondrial H_2_O_2_

In this study, we systematically assessed how loss of the mitochondrial and cytosolic PRDXs affects H_2_O_2_ dynamics in both cytosol and mitochondria and impacts cellular fitness. We confirmed the particular importance of PRDX1 over PRDX2, and PRDX3 over PRDX5 for cytosol and mitochondria, respectively. This fits well with the reported rate constants for the initial reactions with H_2_O_2_ as well as the rates of the reductive half reactions, and the concentrations of the enzymes [23,[48], [49], [50], [51]]. Interestingly, we found that cytosolic PRDXs, in particular PRDX1, exert a dramatic impact on the mitochondrial H_2_O_2_ environment. Loss of PRDX1 unbalances not only cytosolic but also mitochondrial matrix redox homeostasis, and results in deleterious effects for cellular fitness in acute and chronic exogenous and chronic mitochondrial H_2_O_2_ stress experiments. While its impact on handling exogenous H_2_O_2_ pulses might be easily explained by the fact that this H_2_O_2_ has to traverse the cytosol on its way to mitochondria, its effects on mitochondrion-generated H_2_O_2_ is more difficult to explain. PRDX1 and PRDX3 exhibit similar initial reaction rates with H_2_O_2_ that render both of them highly efficient H_2_O_2_ sentinels [[52], [53], [54], [55]]. For both, the reducing reactions are orders of magnitudes slower and considered to be limiting in the catalytic cycle implying rapid oxidation and “inactivation” of the enzymes if H_2_O_2_ formation persists [56]. However, the cytosolic 2-Cys peroxiredoxins (PRDX1/2) appear to be much more abundant than the mitochondrial ones (PRDX3/5) [57]. We thus propose that after the rapid initial overwhelming of matrix PRDXs in cases of pathophysiological generation rates of H_2_O_2_ in the matrix (*e.g.* during reverse electron transfer through complex I), H_2_O_2_ becomes released from mitochondria and reduced in the cytosol. This would be further supported by the higher peroxide concentrations in the matrix of fission yeast and higher eukaryotes compared to the cytosol even at basal levels [58,59]. The formation of steep gradients of H_2_O_2_ originating from mitochondria by extramitochondrial antioxidant systems further supports the continuous and rapid release of H_2_O_2_ [22,[59], [60], [61]]. This “extramitochondrial” handling of mitochondrial H_2_O_2_ is not only limited to cytosolic PRDXs but has also recently been reported to involve peroxisomes, which are for this purpose in contact with mitochondria [62]. Moreover, oxidative burst mechanisms to eject H_2_O_2_ from cristae were reported as mode to allow H_2_O_2_ signaling [63]; however, such a mechanism might also help rapid H_2_O_2_ release during detoxification and establish the cytosol as the sink that “empties mitochondria” from H_2_O_2_.

Membranes pose significant barriers for H_2_O_2_ transport that can lead to a drop of H_2_O_2_ concentrations by 3-to-4 fold [64]. This might be further increased during oxidative stress that has been proposed to remodel membranes and decrease their permeability [65,66]. Together with a high antioxidative activity in one compartment, gradients of more than a hundred-fold can be established. Our modelling and experimental approaches indicate the IMM to be highly permeable towards H_2_O_2_ likely by at least one order of magnitude more compared to the PM. This high permeability could stem from the presence of dedicated H_2_O_2_ transporters as they have been described for signalling at the PM [15,67,68]. For the OMM, porins or VDACs (voltage-dependent anion channels) have been demonstrated to support H_2_O_2_ release [69,70]. For the IMM no strong evidence for dedicated H_2_O_2_ transporters exists. Aquaporins (AQP; in the context of H_2_O_2_ transport also referred to as peroxiporins) have previously been suggested to be present in the IMM, however solid evidence for their IMM localization is missing [34,71,72]. One of the aims of our CRISPR screen was thus to obtain evidence for possible H_2_O_2_ transporters in the IMM. Our sgRNA library included sgRNAs directed against the genes for AQP10, AQP11, AQP12/12A/X2, AQP12B, AQP2, AQP3, AQP4, AQP5, AQP6, AQP7/7L, AQP8, AQP9. However, none of them was among the hits. This either points to them not being critical in the context of mitochondrial H_2_O_2_ homeostasis (*e.g.* because direct membrane diffusion is dominant) or the presence of multiple AQPs in mitochondria that might compensate for each other's loss. Instead, we identified seven enriched and depleted sgRNAs which target genes encoding IMM proteins (depleted sgRNAs: PHB2, BDH1; enriched sgRNAs: SLC25A29, COQ6, DMAC2, CYP24A1, IMMT). Of those proteins, SLC25A29 is an IMM transporter, which was originally reported to be a mitochondrial carnitine-acylcarnitine-like translocase [73] or an ornithine transporter [74], and later characterized as a mitochondrial transporter of basic amino acids, with a preference for arginine and lysine [75]. Interestingly, SLC25A29 has been reported to increase mitochondria-derived nitric oxide due to its role in arginine transport [76]. Both, PRDX3 and PRDX5 were implicated in handling peroxynitrite, the product of the reaction of nitric oxide with superoxide anions [77,78]. Loss of SLC25A29 might thus supress peroxynitrite generation and be protective.

### 3.2 Mitophagy serves as mechanism to compensate for the loss of cytosolic PRDX activity during chronic mitochondrial oxidative stress

Instead of mitochondrial H_2_O_2_ export mechanisms, we identified mitophagy (potentially autophagy) as a major contributor to compensate for the loss of cytosolic PRDXs. We find mitophagy to be important already at unperturbed conditions in PRDX1/2 DKO cells for which we show increased oxidized HyPer7 BLs, and already at IC_10_ D-Ala concentrations H_2_O_2_ can be detected in the cytosol.

A previous genome-wide screen assessing handling of exogenous bolus H_2_O_2_ [79] identified as major rescue mechanisms increased cytosolic NADPH regeneration by loss of the third enzyme of the pentose phosphate pathway, and a better handling of cytosolic H_2_O_2_ by catalase, a peroxisomal protein which accumulated in the cytosol if components of the peroxisomal import machinery were lost. None of these genes were targeted by our mitochondrion-focused screen. This previous study did not observe a particular prominent effect of mitophagy indicating that this process might become particularly prominent for mitochondrial H_2_O_2_ stress.

A different study using rather high millimolar amounts of bolus exogenous H_2_O_2_ also observed increased mitophagic flux [43]. In their case, like in ours it remained unclear how mitochondrial H_2_O_2_ stress is sensed and used to initiate mitophagy. Another study reported mitophagic clearance upon oxidative stress during hypoxia [80]. Oxidation can influence mitophagy at multiple points, for instance, directly through FIS1, which, when oxidized, increases mitochondrial fission via DRP1 recruitment, or indirectly via increased translocation of RAB5 in response to oxidative stress [81,82]. DRP1, in addition, was also identified as being activated in response to mild oxidative stress, thereby promoting mitochondrial fragmentation and facilitating the removal of damaged mitochondria.

Collectively, our study reveals mechanisms that maintain mitochondrial H_2_O_2_ homeostasis across different redox regimes. Under basal conditions, mitochondrial PRDX3 efficiently prevents the accumulation of matrix H_2_O_2_, although even in this state cytosolic PRDXs measurably contribute to shaping matrix H_2_O_2_ levels. When mitochondrial H_2_O_2_ flux increases modestly, cytosolic PRDXs become the dominant regulators of mitochondrial H_2_O_2_ by establishing steep H_2_O_2_ gradients across the inner mitochondrial membrane and promoting continuous H_2_O_2_ efflux for cytosolic clearance. If this buffering system becomes insufficient, mitophagy serves as a backup pathway to preserve organellar integrity during chronic oxidative stress (Fig. 6H).

## 4 Materials and methods

For cell lines, plasmids, primers, antibodies, chemicals, and software and algorithms used in this study, see Supplementary Tables S1–S6, respectively.

### 4.1 Plasmids and cell lines

All cell lines were cultivated using Dulbecco's modified Eagle's medium (DMEM) complete containing 4.5 g/l glucose, 7% (v/v) fetal bovine serum (FBS), 1% (v/v) penicillin and streptomycin (P/S), 1 mM sodium-pyruvate (100 mM), 1x MEM non-essential amino acids (100x) and 50 μg/mL uridine (50 mg/mL)).

### 4.2 Generation of knockout cells

Guide RNA sequences targeting human PRDXs, namely PRDX3, PRDX5 and PRDX6 were cloned into the pSpCas9(BB)‐2A‐GFP (PX458) vector. Guide RNA sequences targeting TXNRD2 were cloned into the pSpCas9(BB)-2A-Puro (PX459) V2.0 vector. Both vectors were a gift from Feng Zhang (Addgene plasmid # 48138, # 62988) [83]. HEK Flp‐In™ T‐REx™‐293 cells were transfected using PEI. Cells transfected with the GFP construct were sorted via FACS, approximately 24 h after transfection. Cells transfected with the Puromycin resistant construct were exposed to puromycin for 2 days, approximately 24 h after transfection. GFP-positive and Puromycin-resistant cells were collected and were single cell seeded on a 96-well plate. Clones were screened using Western blot analysis.

Stable inducible cell lines expressing mtDAO and OMMDAO were generated via the Flp‐In™T‐REx™ system (Invitrogen). For selection of clones, DMEM complete supplemented with 100 μg/ml of hygromycin and 10 μg/ml of blasticidin was used. Inducibility of clones was screened using Western blot analysis. Prior to experiments, the expression of mtDAO and OMMDAO was induced for at least 16 h with 1 μg/ml of doxycycline.

### 4.3 Steady state protein levels

HEK239 cells grown in glucose DMEM complete were harvested in 1x Laemmli buffer (2% SDS, 60 mM of Tris–HCl pH 6.8, 10% glycerol, 0.0025% bromophenol blue) with 50 mM DTT, boiled at 96 °C for 5 min and subsequently analysed by SDS‐PAGE and Western blot analysis. 2,2‐trichloroethanol (TCE) was added to the SDS-PAGE gel to quantify the protein concentration of each sample.

### 4.4 Quantitative label‐free proteomics

HEK293 cells grown in either glucose or galactose DMEM complete were seeded on 6‐well plates (n = 4). Confluency was closely monitored and when reaching 80% confluence, cells were washed once with 1x PBS, and collected in 1 ml of 1x PBS in a 1.5‐mL low-binding reaction tube. Cells were pelleted by centrifugation at 300×*g* for 7 min, supernatant was removed, and lysis buffer (1x PBS, 4% SDS, containing 1x protease inhibitor cocktail) was added. To degrade the chromatin, the samples were sonicated (20x 30/30) and boiled at 96 °C for 5 min to precipitate proteins. A fourfold volume of ice-cold acetone was added, and samples were frozen overnight at −80 °C. The next day, samples were thawed on ice and centrifuged for 15 min at 16.000×*g* at 4 °C. The pellets were washed twice with ice-cold acetone and air-dried. Cell pellets were lysed in Urea buffer (8 M Urea and 50 mM TEAB containing 1x protease inhibitor cocktail) and sonified (15x 30/30). Protein concentration was determined and 50 μg of protein was reduced by the addition of 5 mM DTT at 37 °C for 1 h. Afterwards, 40 mM chloroacetamide (CAA) was added to the sample and incubated for 30 min at RT in the dark. The in-solution digest was performed by adding Lys-C protease (1:75) for 4 h at 25 °C. The sample was further digested overnight at 25 °C by Trypsin (1:75) after dilution of the urea concentration to <2 M. Digestion was terminated by the addition of 1% formic acid. Samples were loaded onto a two-layer SDB-RPS stagetip, which were used for desalting/mixed-phase clean up. Mass spectrometry was performed by the proteomics core facility from CECAD Cologne, followed by data processing and initial analyses. Prepared samples were loaded on a Q Exactive TM Plus Orbitrapmass spectrometer coupled to an EASY nLC (both Thermo FisherScientific).

The data was further analysed using R pipelines. Data was assessed for quality, significance and GO-term analysis.

### 4.5 HyPer7 measurements

Measurements were done as described in Ref. [30]. Specifically, four and a half thousands cells/well were seeded on a poly-l-lysine-coated 96 well plate (μclear, GreinerBio). Approximately 24 h after seeding, transient transfection of the respective sensors was performed using PEI. If cells were expressing the mtDAO and OMMDAO constructs, expression was induced 24 h before the measurement by addition of 1 μg/ml of doxycycline.

Approximately 48 h after transient transfection, DMEM complete medium was replaced by minimal medium (containing 140 mM NaCl, 5 mM KCl, 1 mM MgCl_2_, 2 mM CaCl_2_, 20 mM Hepes, 10 mM glucose, pH set to 7.4 with NaOH). The plate was incubated for 30 min in the Cytation 3 (BioTek) at 37 °C and 5% CO_2_. All measurements were performed by utilizing the 10x in-air microscope and the BioTek LED filter cubes 390 ± 9 nm and 469 ± 17.5 nm. First, a 30 min steady state was imaged, followed by treatment of the plate which was followed for an additional 60 min. To calibrate the sensor responses, the wells were subsequently treated with oxidation (20 min) followed by reduction (20 min) of the sensor.

Cells were either treated with a bolus of H_2_O_2_ or D/l-Alanine (if cells were expressing the DAO tool). Finally, 20 μM H_2_O_2_ was added followed by 5 mM DTT as sensor oxidizing and reducing control.

The acquired images for each experiment were analysed using the program RRA (“redox ratio analysis”; [84]. With this program, images were aligned, filtered, background‐subtracted, and the intensity for both channels as well as the resulting ratio (469/390 or 390/469) was calculated and saved as an excel file. Using R, these excel files were further analysed, the mean was calculated, figures prepared, and statistics performed. Data was represented in various ways; 1) response curves over time, mean value with the standard deviation, 2) steady state, boxplot visualising the median value of single cells over 10 min before treatment, 3) AUC, boxplot visualising the median value of single cell AUCs. Curves were normalized to the steady state and AUC was calculated over a time period of 40 min. For Cytation3 measurements a Wilcoxon/Mann–Whitney‐U‐test was performed and samples were compared in pairs.

### 4.6 Cell proliferation assay to test for growth on different carbon sources

Cell proliferation was assessed with the Omni™ live-cell analysis platform, through the monitoring of cell confluency through brightfield analysis. Cell lines were seeded on a poly-L-coated 48-well plate at a density of 15.000 cells/well in replicates of 8 and incubated at 37 °C. Approximately 10 h after seeding, the plate was transferred to the Omni™ platform and measurement was started for a duration of 7 days. Approximately 24 h after seeding the medium was exchanged with DMEM containing glucose or galactose (DMEM supplemented with 4.5 g/l glucose or galactose, 1 mM sodium pyruvate, 1x non-essential amino acids, 7% FBS and 50 μg/ml Uridine). Every day the medium was exchanged by removing 250 μl and adding 250 μl fresh DMEM containing either glucose or galactose.

The captured pictures were analysed by the Omni™ confluency module, after which the data could be exported and visualized with graphs based on a confluency percentage. To calculate the AUC, an R pipeline was set-up to which took the seeding confluency into account.

### 4.7 Viability assay

Cells were seeded at a confluency of 10.000 cells/well on a poly-l-lysine-coated 96-well plate. Cells were incubated for 48 h before exposure to the various treatments. In regard to mtDAO viability assessment 24hrs. before treatment, expression was induced by addition of 1 μg/ml of doxycycline.

Cells were either treated with H_2_O_2_, d-Alanine (if cells were expressing the mtDAO tool) or VPS34-IN-1. After the incubation period of either 5, 24 or 48 h, the medium was carefully aspirated from the cells, and 100μl/well viability media (DMEM complete containing 4.5 g/l glucose, 7% (v/v) FBS and 10 % (v/v) PrestonBlueTM) was added without detaching the cells. The plate was thereafter incubated in the dark for 1.5 h at 37 °C with 5 % CO_2_. The fluorescent formation of resorufin was measured at 560 nm (excitation) and 590 nm (emission) using the ClarioStar (BMG). The measured fluorescent intensities were first blank-subtracted, where after the values were compared to the 100 % viability control. Statistical analysis was performed using the Welch's *t*-test to compare the WT with each cell line separately.

### 4.8 Ferroptosis cell death assay

50.000 cells/well were plated in 24-well plates and treated with the GPX4 inhibitor RSL3 (Selleck Chem) at increasing concentrations indicated. After 8 h, cells were washed, collected and pelleted by centrifugation for 5 min at 1200 rpm. To quantify cell death, the cell pellet was then resuspended in 200 μl of 1x PBS supplemented with 2% FBS and 1 μg/ml propidium iodide (PI). The PI fluorescent signal was acquired on the BD LSRFortessa (BD Biosciences) and analysed with Flowjo V10.6.2.

### 4.9 Mitophagy assay using mKeima

Cells were seeded on a 10 cm dish with a density of five million cells. The next day, cells were transfected with 5/10 μg of plasmid DNA, containing the mKeima constructs, by GeneJuice (according to the manufacturer's protocol). 24 hrs after transfection, cells were collected and seeded at a density of one million cells/well on a 6-well plate. Simultaneously, expression of the mtDAO construct was induced by the addition of 1 μg/ml of doxycycline. On the fourth day, cells were treated with D- and L-Ala for 8 h, after which the cells were washed and pelleted by centrifugation at 500×*g* for 3 min. As a positive control, cells were treated for 6-8 h with 10 μM oligomycin and 10 μM antimycin A with or without 100 mM bafilomycin. The cell pellet was thereafter resuspended in FACS buffer (10% FBS in 1x PBS). Samples were kept on ice and analysed directly using FACS. Fluorescent intensities of mKeima were measured using a BD LSRFortessa (BD Biosciences) with excitation at 405 nm (acidic form) and 561 nm (neutral/basic form), and emission collected through a 610/20 nm bandpass filter. The data were analysed using FlowJo v10.10.0. Events were gated as follows: FSC-A vs. SSC-A to exclude debris, FSC-W vs. FSC-A to select singlets, and mKeima acidic and neutral/basic fluorescence to define the transfected population (using untransfected, doxycycline-treated cells as negative control). Within the transfected gate, mitophagy was quantified as the mean 561/405 excitation ratio. A minimum of 8000 events per sample were analysed.

### 4.10 GSx assay

The assay was done as described in Ref. [85]. Specifically, twenty-five thousand cells per well were seeded on a poly-l-lysine-coated 24-well plate. An experimental plate was always seeded in duplicate to enable accurate protein determination. Approximately 4 h before the experiment, the media was exchanged to DMEM complete (containing 4.5 g/l glucose, without FBS and antibiotics). The cells were washed with ice-cold 1x PBS, and thereafter lysed with 1 % (w/v) sulfo salicylic acid (SSA) solution. Before quantitatively transferring the samples, the experimental plate was first incubated on ice for 10 min. The sample was cleared by centrifugation at 15.000×*g* for 1 min. To determine the GSx concentration, a 96-well plate format was used, in which each sample was measured with 6 technical replicates. Samples were prediluted 1:10 with dH_2_O, and 100 μL was transferred into each well. The reaction was initiated by the addition of 100 μL reaction buffer (50 mM NaPi, pH 7.5, containing 0.5 mM EDTA, 0.2 mM NADPH, 0.15 mM DTNB, 0.1 U yeast glutathione reductase (GR)). The formation of TNB was monitored for 15 min, with measurements taken approximately every 30 s. A GSx standard was included on each measured plate to enable correlation with GSx concentrations. The rate of formation of TNB is proportional to the GSx content, linear regression is used to determine the total GSx content and is normalized to the protein concentration of the second 24-well plate. For protein determination of the second experimental plate, cells were lysed using the RIPA Lysis method. Statistical analysis was performed by utilizing the Student's T-test assuming unequal variances.

### 4.11 Modelling

Modelling was performed as explained in Supplementary Methods S1 – S4 based on values presented in Supplementary Tables S7 and S8.

### 4.12 CRISPR drop-out screen

The screen was done as described in Ref. [86]. Selection of genes and design of sgRNA library was performed as described in Supplementary Methods S5 and S6. The two cell lines of interest were seeded on 15 cm dishes according to a 200-fold coverage of the screen, considering the MOI of 30 % and the number of conditions (neg. ctr (T0), L-Ala, D-Ala (IC10, IC50, IC90)). Simultaneously with the seeding, the produced virus was added with 5 μg/mL protamine. Plates were placed at 37 °C with 5 % CO_2_ for 24 h before selection was started. 1 μg/mL puromycin was added to each plate after 24 h and selected for 24 – 48 h, depending on the negative control.

The selected cells from each cell line were thereafter detached and pooled before seeding for the drop-out screen. The pooled cells were counted and seeded with a 200fold coverage of the screen per condition. The T0 was directly collected and stored at 80 °C. A day after seeding, induction of the mtDAO tool was started by the addition of 1 μg/mL Dox to each plate. After 24 h, the cells were treated either with L-Ala (Ctr) or D-Ala with various concentrations (IC10, IC50, IC90). After 48 h, the cells were detached and collected by centrifugation at 300×*g* for 5 min. The cell pellet was washed and stored at −80 °C.

Genomic DNA was isolated from the samples prepared for sequencing on a NextSeq 1k platform (Illumina, Inc.), see Supplementary Table S9 for barcode sequences. After demultiplexing of the data, the overall quality of the FASTQ files were checked with help of the MultiQC program (v1.11); https://www.bioinformatics.babraham.ac.uk/projects/fastqc/.

### 4.13 Extracting gRNAs from sequencing reads and statistical analysis of gRNAs

Using the FASTQ Filter Module (FFM) from the ENCoRE tool [87] identified all 26 base single-end reads within a FASTQ file. Each sequencing read was cropped directly after 20 bp from the beginning of the sequence. The remaining bases were discarded. The residual 20 bp sequences correspond to the CRISPR guide RNA (gRNA) sequences from the CRISPR knockout library. The filtered FASTQ files and a comma-separated (csv) reference file containing the gene annotation of each gRNA **Supplemental Dataset S2** were entered into the MAGeCK program [38] to count gRNAs using the subcommand “mageck count”. The generated read count table was used to compare different treatment and control conditions using the subcommand “mageck test” with the parameter “--norm-method total” and to determine negatively and positively selected genes as well as the ranking of gRNAs. Gene log fold changes (LFC) from gRNA LFCs were calculated by choosing the parameter “--gene-lfc-method mean” in the “mageck test” subcommand.

Following the initial MAGeCK analysis, the data were further processed in R. A p-value threshold of <0.05 and a log fold change (LFC) of < −1 or >1 were used to filter for potential hits. The filtered data was visualized using volcano plots. A GO term was in addition also performed using R, and visualized using boxplots.

## Declaration of generative AI and AI-assisted technologies in the manuscript preparation process

During the preparation of this work, the authors used ChatGPT in order to improve the readability and language of the manuscript. After using this tool, the authors reviewed and edited the content as needed and take full responsibility for the content of the published article.

## CRediT authorship contribution statement

**Lianne JHC. Jacobs:** Conceptualization, Data curation, Formal analysis, Investigation, Methodology, Validation, Visualization, Writing – original draft, Writing – review & editing. **Sebastian Doll:** Investigation, Methodology, Validation, Writing – review & editing. **Dietrich Trümbach:** Data curation, Formal analysis, Methodology, Software, Writing – review & editing. **Matteo Veronese:** Formal analysis, Investigation, Methodology, Validation, Writing – review & editing. **Giada Di Pietro:** Formal analysis, Investigation, Methodology, Validation, Writing – review & editing. **Fatma Isil Yapici:** Formal analysis, Investigation, Methodology, Validation, Writing – review & editing. **Lidwina Hasberg:** Formal analysis, Investigation, Methodology, Validation. **Pascal Gentzsch:** Formal analysis. **Sarah Gerlich:** Resources. **Jens Hansen:** Resources. **Silvia von Karstedt:** Supervision, Writing – review & editing. **Elena I. Rugarli:** Supervision, Writing – review & editing. **Marcus Conrad:** Funding acquisition, Supervision, Writing – review & editing. **Armindo Salvador:** Conceptualization, Investigation, Methodology, Software, Visualization, Writing – review & editing. **Jan Riemer:** Conceptualization, Formal analysis, Funding acquisition, Project administration, Resources, Supervision, Validation, Visualization, Writing – original draft, Writing – review & editing.

## Declaration of competing interest

Marcus Conrad is a co-founder and shareholder of ROSCUE Therapeutics GmbH. All other authors have nothing to disclose and no conflict of interest.

## Data Availability

Data will be made available on request.
