# Supplementary material for: Cytosolic PRDX1 acts as an extramitochondrial sink to set mitochondrial H2O2 levels and enable resilience to chronic mitochondrial oxidative stress
Source: Redox Biol. 2026 Apr 30;94:104195. doi: 10.1016/j.redox.2026.104195 (PMC13158620; doi:10.1016/j.redox.2026.104195)
Supplement: Multimedia component 4 [file mmc4.pdf]

SUPPLEMENTAL INFORMATION

Cytosolic PRDX1 acts as an extramitochondrial sink to set mitochondrial H_2O_2 levels and enable resilience to chronic mitochondrial oxidative stress

Lianne JHC Jacobs[1,2], Sebastian Doll[3], Dietrich Trümbach[3], Matteo Veronese[2,4], Giada Di Pietro[2,4], Fatma Isil Yapici[2,5], Lidwina Hasberg[1,2], Pascal Gentzsch[1,2], Sarah Gerlich[1,2], Jens Hansen[3], Silvia von Karstedt[2,5], Elena I. Rugarli[2,4,6], Marcus Conrad[3,7], Armindo Salvador[8,9,10,11], Jan Riemer[1,2,6,*]

1, Redox Metabolism Group, Institute for Biochemistry, University of Cologne, 50674 Cologne, Germany

2, Cologne Excellence Cluster on Cellular Stress Responses in Aging-Associated Diseases (CECAD), University of Cologne, 50931 Cologne, Germany.

3, Helmholtz Zentrum München, Institute of Metabolism and Cell Death, 85764 Neuherberg, Germany.

4, Institute for Genetics, University of Cologne, 50674 Cologne, Germany

5, Department of Translational Genomics, Faculty of Medicine and University Hospital Cologne, University of Cologne, 50931 Cologne, Germany.

6, Center for Molecular Medicine, University of Cologne

7, Translational Redox Biology, Technical University of Munich (TUM), TUM Natural School of Sciences, 85748 Garching, Germany

8, CNC-UC - Centre for Neuroscience Cell Biology, University of Coimbra, 3004-504 Coimbra, Portugal

9, CiBB - Centre for Innovative Biomedicine and Biotechnology, University of Coimbra, 3004-504 Coimbra, Portugal

10, Coimbra Chemistry Center - Institute of Molecular Sciences (CQC-IMS), University of Coimbra, 3004-535 Coimbra, Portugal

11, Institute for Interdisciplinary Research, University of Coimbra, 3030-789 Coimbra, Portugal

* address correspondence to

J.R.: jan.riemer@uni-koeln.de, +49-221-470-7306, ORCID 0000-0002-7574-8457

Table of content

Supplementary tables

Table S1. Cell lines page 3

Table S2. Plasmids page 4

Table S3. Primers page 4

Table S4. Antibodies page 5

Table S5. Chemicals page 6

Table S6. Software and algorithms page 6

Table S7. Kinetic and concentration parameters used for modelling page 7

Table S8. Logarithmic sensitivities page 9

Table S9. Barcode CRISPR screen page 10

Supplementary methods

Method S1. Assumptions for the modelling page 10

Method S2. Calculation of H_2O_2 generation rates page 12

Method S3. Parameter describing IMM transport of H_2O_2 page 12

Method S4. Sensitivity assay page 13

Method S5. Preselection of genes for the CRISPR knockout library page 14

Method S6. Design of the sgRNA library page 14

Supplementary figures

Figure S1. Characterization of PRDX KO cell lines page 17

Figure S2. Mitochondrial DAO cell lines page 18

Figure S3. Modelling cellular H_2O_2 dynamics Page 20

Figure S4. Viability and ferroptosis analysis of PRDX KO cell lines page 23

Figure S5. Targeted CRISPR drop out screen page 24

Figure S6. Investigation of mitophagy page 27

Supplementary dataset

Dataset 1. Source data Cytation

Dataset 2. sgRNA library annotation

Dataset 3. CRISPR screen sequencing data

Table S1. Cell lines

Cell line	Plasmid	Construct	Tag	Reference
HEK293 Flp-In T-REx WT	-	-	-	Invitrogen Cat# R78007
HEK293 Flp-In T-REx WT + mtDAO	pcDNA5	Su9^{MTS}(1-69)-RgDAO	-FLAG	[1]
HEK293 Flp/IN T-Rex WT + OMMDAO	pcDNA5	TOM20^{MTS}(1-29)-RgDAO	-FLAG	This study
HEK293 Flp-In T-REx PRDX1 KO	-	-	-	[1]
HEK293 Flp-In T-REx PRDX1 KO + mtDAO	pcDNA5	Su9^{MTS}(1-69)-RgDAO	-FLAG	[1]
HEK293 Flp-In T-REx PRDX2 KO	-	-	-	[1]
HEK293 Flp-In T-REx PRDX2 KO	pcDNA5	Su9^{MTS}(1-69)-RgDAO	-FLAG	[1]
HEK293 Flp-In T-REx PRDX1/2 DKO	-	-	-	[1]
HEK293 Flp-In T-REx PRDX1/2 DKO + mtDAO	pcDNA5	Su9^{MTS}(1-69)-RgDAO	-FLAG	[1]
HEK293 Flp/IN T-Rex PRDX1/2 DKO + OMMDAO	pcDNA5	TOM20^{MTS}(1-29)-RgDAO	-FLAG	This study
HEK293 Flp-In T-REx PRDX3 KO	-	-	-	This study
HEK293 Flp-In T-REx PRDX3 KO + mtDAO	pcDNA5	Su9^{MTS}(1-69)-RgDAO	-FLAG	This study
HEK293 Flp-In T-REx PRDX5 KO	-	-	-	This study
HEK293 Flp-In T-REx PRDX5 KO + mtDAO	pcDNA5	Su9^{MTS}(1-69)-RgDAO	-FLAG	This study
HEK293 Flp-In T-REx PRDX3/5 DKO	-	-	-	This study
HEK293 Flp-In T-REx PRDX3/5 DKO + mtDAO	pcDNA5	Su9^{MTS}(1-69)-RgDAO	-FLAG	This study
HEK293 Flp-In T-REx PRDX6 KO	-	-	-	This study
HEK293 Flp-In T-REx GSR KO	-	-	-	[2]
HEK293 Flp-In T-REx TXNRD2 KO	-	-	-	This study
HEK293T	-	-	-	ATCC CRL-3216

Table S2. Plasmids

Plasmid	Backbone	Insert	Reference
Cyto-HyPer7	pCS2+	HyPer7-RevNES(3x)	[3]
Matrix-HyPer7	pCS2+	2xCox8$^{MTS(1\text{-}25)}$ -HyPer7	[3]
mtDAO	pcDNA5/FRT/TO	Su9$^{MTS(1\text{-}69)}$-RgDAO-FLAG	[1]
OMMDAO	pcDNA5/FRT/TO	TOM20$^{MTS(1\text{-}29)}$-RgDAO-FLAG	This study
mt-mKeima	mKeima-Red	COX8-MTS	mKeima-Red-Mito-7 was a gift from Michael Davidson (Addgene plasmid # 56018)
mKeima-FIS1^{MTS}	pB-UbC-ΔCuO- and mKeima-Red-Mito-7	FIS1 C-terminal sequence (101-152)	E. Rugarli (A. Kaczmarek, A. Lefèvre)
PRDX3 CRISPR	pspCas9(BB)-2A-GFP (PX458)	PRDX3 gRNA	This study
PRDX5 CRISPR	pspCas9(BB)-2A-GFP (PX458)	PRDX5 gRNA	This study
PRDX6 CRISPR	pspCas9(BB)-2A-GFP (PX458)	PRDX6 gRNA	This study
TXNRD2 CRISPR	pspCas9(BB)-2A-Puro (PX459) V2.0	TXNRD2 gRNA	This study
psPAX2	psPAX2	-	M. Conrad
pMD2.G	pMD2.G	-	M. Conrad
CRISPY/CRISP library	CRISPY/CRISP	gRNA library	This study

Table S3. Primers

Oligo	Sequence (5' – 3')	Purpose
PRDX3 G29 fwd	CACCGCTCGTTCTTCCACATGCAGC	PRDX3 KO generation
PRDX3 G29 rev	AAACGCTGCATGTGGAAGAACGAGC	PRDX3 KO generation
PRDX5 G8 fwd	CACCGTTGGGAATCGACGTCTCAAG	PRDX5 KO generation
PRDX5 G8 rev	AAACCTTGAGACGTCGATTCCCAAC	PRDX5 KO generation
PRDX6 G3 fwd	CACCGACACTGGGGTAAAGTCCCGA	PRDX6 KO generation
PRDX6 G3 rev	AAACTCGGGACTTTACCCCAGTGTC	PRDX6 KO generation

TXNRD2 G4 fwd	CACCGCACTACCTGGTCGAAGCCGCGG	TXNRD2 KO generation
TXNRD2 G4 rev	AAACCCGCGGCTTCGACCAGGTAGTGC	TXNRD2 KO generation
LCV2 fw	GAGGGCCTATTTCCCATGATTC	Enriching gRNA
LCV2 rev	GTTGCGAAAAAGAACGTTCACGG	Enriching gRNA
DAO fw	CGCGGATCCCACAGCCAGAAGAGGGTG	OMMDAO generation
DAO rev	GCGGCGGCCGCTCACTTGTCGTCATCGTCTTTGTAGTCAGATCCGCTCTCCCTAGC	OMMDAO generation
TOM20 fw	TTAAGGCCACCATGGTGGGTCGGAACAGCGCCATCGCCGCCGGTGTATGCGGGGCCCTTTTCATTGGGTACTGCATCTACTTCGACCGCAAAAGACGAGGGGGGTCCGGGG	OMMDAO generation
TOM20 rev	GATCCCCCGGACCCCCCTCGTCTTTTGCGGTCGAAGTAGATGCAGTACCCAATGAAAAGGGCCCCGCATACACCGGCGGCGATGGCGCTGTTCCGACCCACCATGGTGGCC	OMMDAO generation

Table S4. Antibodies

Antibody	Source	Identifier
PRDX1 - Polyclonal antibody (Rabbit)	Sigma-Aldrich	HPA007730
PRDX2 - monoclonal antibody (Mouse)	Sigma-Aldrich	WH0007001M1
PRDX3 - Polyclonal antibody (Rabbit)	Sigma-Aldrich	HPA041488
PRDX5 - Polyclonal antibody (Rabbit)	Sigma-Aldrich	HPA037915
SDHA - Monoclonal antibody (Mouse)	Invitrogen	459200
Actin - Monoclonal antibody (Mouse)	ThermoFisher Scientific	MA5-11869
LDH (H-10) – Monoclonal antibody (Mouse)	Santa Cruz	Sc-133123
LC3B – Monoclonal antibody (Rabbit)	ABclonal	A19665
Flag M2 - Monoclonal antibody (Mouse)	Sigma-Aldrich	F3165
Goat αRabbit HRP	ImmunoReagents	GtxRb-003-DHRPX
Goat αMouse HRP	ImmunoReagents	GtxMu-003-DHRPX

Table S5. Chemicals

Chemical/ reagent	Company	Identifier
Hydrogen Peroxide (H_2O_2) solution, 30 wt. % in H_2O	Sigma Aldrich	216763-100ML
PrestoBlue Cell viability reagent	Thermo Fisher Scientific	A13262
ROTI®Quant universal	Carl Roth	0120.1
Propidium Iodide (PI)	Sigma Aldrich	P4170-10MG
RSL3	Selleck Chem	S8155
Glutathione Reductase from baker's yeast (S. cerevisiae)	Merck	G3664-500UN
5,5'-Dithio-bis(2-nitrobenzoic acid) 99% (DTNB)	Aldrich Chemistry	D21,820-0
NADPH, approx. 98%	Roche	10107824001
D-Alanine	Sigma Aldrich	338-69-2
L-Alanine	AppliChem	A3690,0100
Diamide	Sigma Aldrich	10465-78-8
DTT	AppliChem	A1101,0025
Oligomycin	Sigma Aldrich	O4876-5MG
Antimycin A	Sigma Aldrich	A8674-100MG
VPS34-IN-1	Hycultec	HY-12795

Table S6. Software and algorithms

Software/ Algorithm	Reference
Image Lab 5.2	https://www.bio-rad.com/
Redox ratio analysis "RRA"	Fricker, 2016 [4]
R	https://www.r-project.org/
Rstudio IDE	https://www.rstudio.com/
BioTek Gen5	https://www.agilent.com/
FlowJo V10.6.2 software	https://www.flowjo.com/
GraphPad Prism 9	https://www.graphpad.com/scientific-software/prism/
MAGeCK program	http://liulab.dfci.harvard.edu/Mageck
Gene Ontology	www.amigo.geneontology.org/amigo/search/bioentity
MitoCarta2.0	www.broadinstitute.org/files/shared/metabolism/mitocarta/human.mitocarta2.0.html
IMPI	www.mrc-mbu.cam.ac.uk/research-resources-and-facilities/impi
Mathematica	Wolfram Research Inc., Mathematica, Version 14, Wolfram Research, Inc., Champaign, IL, U.S.A., 2024
DepMap	www.depmap.org/portal/data_page/?tab=allData

Table S7. Kinetic and concentration parameters used for modelling

	Parameter	Value	Units	Description
Parameters from the Stein et al. (2020) model for the mitochondrial matrix	k_1	4	μMs^{-1}	Intracellular peroxide production
	k_2	60	$\mu M^{-1}s^{-1}$	GPx1red reacting with H_2O_2
	k_3	0.04	$\mu M^{-1}s^{-1}$	GPx1ox reacting with GSH
	k_5	57	μM	K_M of $NADP^+$
	k_6	20	$\mu M^{-1}s^{-1}$	PRDX3-SH oxidized by H_2O_2
	k_7	0.014	$\mu M^{-1}s^{-1}$	PRDX3-SOH over-oxidized by H_2O_2
	k_8	0.003	$\mu M^{-1}s^{-1}$	Reduction of overoxidized PRDX3 by sulfiredoxin
	k_9	20	s^{-1}	Self-catalyzed disulfide formation of PRDX3-SS from PRDX3-SOH
	k_{10}	0.22	$\mu M^{-1}s^{-1}$	PRDX3 is reduced by thioredoxin
	k_{11}	0.000074	s^{-1}	Auto-oxidation of GSH
	k_{12}	0.0001	$\mu M^{-1}s^{-1}$	Pr-SH oxidized by H_2O_2
	k_{13}	0.12	$\mu M^{-1}s^{-1}$	Pr-SOH glutathionylated by GSH
	k_{14}	0.012	$\mu M^{-1}s^{-1}$	GRX2-SH de-glutathionylates Protein-SSG
	k_{15}	0.037	$\mu M^{-1}s^{-1}$	GSH de-glutathionylates Grx2-SSG
	k_{17}	0.0001	$\mu M^{-1}s^{-1}$	Pr-SS reduced by Trx
	k_{18}	3.2	$\mu M^{-1}s^{-1}$	GSSG reduced by GR
	k_{19}	20	$\mu M^{-1}s^{-1}$	Oxidized Thioredoxin reduced by TrxR
	k_{20}	375	$\mu M\ s^{-1}$	Regeneration of NADPH
	k_{21}	0.48	$\mu M\ s^{-1}$	GSH import
	k_{22}	0.032	$\mu M\ s^{-1}$	GSH export+degradation
	k_{23}	0.3	$\mu M^{-1}s^{-1}$	PRDX5-SH oxidized by H_2O_2
	k_{24}	14.7	s^{-1}	PRDX5-SOH auto-catalyzes to PRDX5-SS
	k_{25}	2	$\mu M^{-1}s^{-1}$	PRDX5-SS reduced by TRX2
	k_{26}	0.048	$\mu M^{-1}s^{-1}$	GPx4red oxidized by H_2O_2
	k_{27}	0.02	$\mu M^{-1}s^{-1}$	GPx4ox reacting with GSH
	k_{28}	0	$\mu M\ s^{-1}$	DAAO producing H_2O_2
	k_{29}	$1.23\ 10^{-5}$	$\mu M\ s^{-1}$	sulfiredoxin import
	$GPx1_{tot}$	0.015	μM	Total concentration of GPx1 in matrix
	$GPx4_{tot}$	0.23	μM	Total concentration of GPx4 in matrix
	$GRX2_{tot}$	1	μM	Total concentration of Grx2 in matrix
	PrS_{tot}	0.001	μM	Total concentration of protein monothiols in matrix
	$PrSS_{tot}$	1900	μM	Total concentration of protein dithiols in matrix
	Srx_0	0.00878	μM	Initial concentration of sulfiredoxin in matrix
	GSH_0	5000	μM	Initial concentration of reduced glutathione in matrix
	$GSSG_0$	1.78	μM	Initial concentration of oxidized glutathione in matrix
	$NADPH_0$	30	μM	Initial concentration of NADPH in matrix
	$NADP_0$	0.3	μM	Initial concentration of $NADP^+$ in matrix

Table S7, *continued* – Assumption of further parameters

	Parameter	Value	Units	Description
Parameters from the Griffith et al. (2024) model for the cytosol	$k_{Alt,c}$	160	s^{-1}	Alternative cytosolic H_2O_2 sinks
	k_{Se}	100	$\mu M^{-1}s^{-1}$	PRDX1/2-SH sulfenylation
	k_{C1}	11	s^{-1}	PRDX1-SOH condensation
	k_{C2}	0.5	s^{-1}	PRDX2-SOH condensation
	k_{R1}	2.2	$\mu M^{-1}s^{-1}$	PRDX1-SS reduction
	k_{I}	15.8	s^{-1}	PRDX1-SS LU->FF conversion
	k_{R2}	0.61	$\mu M^{-1}s^{-1}$	PRDX2-SS reduction
	k_{Si1}	0.0015	$\mu M^{-1}s^{-1}$	PRDX1-SS sulfinylation
	k_{Si2}	0.0034	$\mu M^{-1}s^{-1}$	PRDX2-SS sulfinylation
	k_{Srx}	0.001	$\mu M^{-1}s^{-1}$	PRDX1/2-SO2H reduction
	V_{Max}	180	$\mu M\ s^{-1}$	Maximal rate of Trx1-SS reduction
	K_M	1.8	μM	K_M of TRX1-SS reduction
	$PRDX1_{tot}$	110	μM	Total concentration of PRDX1 active sites in the cytosol
	$PRDX2_{tot}$	30	μM	Total concentration of PRDX2 active sites in the cytosol
	$TRX1_{tot}$	20	μM	Total concentration of TRX1 in the cytosol

Table S7, *continued* – Assumption of further parameters

	Parameter	Value	Units	Description
New or modified parameters	φ_c	0.2	$\mu M\ s^{-1}$	Non-mitochondrial H_2O_2 supply to the cytosol
	КA	1500	$\mu m^3\ s^{-1}$	Permeability constant x area of the "mitochondrial membrane"
	V_c	1000	μm^3	Volume of cytosol, guestimate
	V_m	20	μm^3	Volume of mitochondrial matrix, guestimate
	$V_{max,TrxR2}$	274	$\mu M\ s^{-1}$	Maximal rate of Trx2 - SS - reduction
	$K_{M,TrxR2,Trx2SS}$	1.8	μM	K_M of TXNRD2 for Trx2 - SS
	$K_{M,TrxR2,NADPH}$	11	μM	K_M of TXNRD2 for NADPH
	$PRDX3_{tot}$	433	μM	Total concentration of PRDX3 active sites in the mitochondrial matrix
	$PRDX5_{tot}$	188	μM	Total concentration of PRDX5 active sites in the mitochondrial matrix
	$TRX2_{tot}$	35	μM	Total concentration of thioredoxin 2 in the mitochondrial matrix
	$TRXR2_{tot}$	15	μM	Total concentration of thioredoxin reductase 2 in the mitochondrial matrix

Table S8. Logarithmic sensitivities of the lowest value of *KA* (*KA*$_{min}$) consistent with PRDX1/2 DKO having a greater influence on the concentration of H_2O_2 in the mitochondrial matrix than the PRDX3/5 DKO. Sensitivities with absolute values higher than 0.1 are italicized.

Parameter	Sensitivity	Parameter	Sensitivity
k_{DAAO}	*-1.40E+00*	$PrSS_{tot}$	-1.19E-06
k_5	*-9.04E-01*	k_{SRX}	-3.26E-07
$K_{M, TRXR2, NADPH}$	*-2.97E-01*	V_{Max}	-7.59E-08
ϕ_c	-7.03E-02	k_{18}	-6.14E-08
k_1	-2.88E-02	k_4	-9.69E-09
$PRDX5_{tot}$	-2.82E-02	k_{27}	-7.86E-11
k_{23}	-2.81E-02	k_{12}	-2.56E-11
k_{Se}	-2.10E-02	PrS_{tot}	-2.50E-11
$PRDX1_{tot}$	-1.57E-02	k_{14}	-2.47E-12
$K_{M, TRXR2, Trx2SS}$	-1.14E-02	k_{13}	-4.65E-13
k_{11}	-6.29E-03	$GRX2_{tot}$	-3.12E-13
GSH_0	-4.75E-03	k_{19}	-2.03E-15
$PRDX2_{tot}$	-4.23E-03	k_{15}	7.54E-13
k_{21}	-1.64E-03	k_{Si1}	2.96E-08
k_{C2}	-3.10E-04	K_M	7.25E-08
$GPX1_{tot}$	-2.32E-04	k_{Si2}	3.75E-07
k_2	-2.28E-04	SRX_{m0}	7.72E-07
k_{C1}	-5.67E-05	k_{29}	2.59E-06
k_I	-3.94E-05	k_8	3.30E-06
k_9	-3.31E-05	k_7	4.29E-05
$TRX1_{tot}$	-2.73E-05	k_{22}	1.09E-04
k_{24}	-2.63E-05	k_6	2.73E-04
k_{17}	-2.51E-05	k_{10}	5.61E-04
k_{R1}	-1.42E-05	$PRDX3_{tot}$	1.19E-03
k_{R2}	-1.30E-05	$TRX2_{tot}$	1.22E-02
k_{25}	-5.37E-06	$V_{max, TRXR2}$	*5.42E-01*
$GPX4_{tot}$	-2.87E-06	$k_{Alt,c}$	*1.05E+00*
k_{26}	-2.87E-06	$NADPH_0$	*1.20E+00*
k_3	-2.41E-06	k_{20}	*1.21E+00*
k_{16}	-2.32E-06		

Table S9. Barcode CRISPR screen

Cell line	Sample	Barcode sequence
WT + mtDAO	T0	ATTACTCG + AGGCTATA
	L-Alanine	ATTACTCG + GCCTCTAT
	D-Alanine IC10	TCCGGAGA + GCCTCTAT
	D-Alanine IC50	GAGATTCC + GCCTCTAT
	D-Alanine IC90	ATTCAGAA + GCCTCTAT
PRDX1/2 DKO + mtDAO	T0	TCCGGAGA + AGGCTATA
	L-Alanine	ATTACTCG + AGGATAGG
	D-Alanine IC10	TCCGGAGA + AGGATAGG
	D-Alanine IC50	GAGATTCC + AGGATAGG
	D-Alanine IC90	ATTCAGAA + AGGATAGG

Method S1. Multi-compartmental modelling of H_2O_2 metabolism

To help rationalize the experimental results we set up a multicompartment kinetic model of H_2O_2 metabolism. To this effect we coupled a slightly modified version of the model in Stein *et al.* (2020) [5] for H_2O_2 metabolism in the mitochondrial matrix to the reaction component (*i.e.*, without diffusional restrictions) of the model in Griffith *et al.* (2024) [6] for H_2O_2 metabolism in the cytosol. We refer the reader to the original publications for the full details of these models and discuss below how they were coupled and modified for the purposes of this work.

The two models were coupled through the permeation of H_2O_2 between the mitochondrial matrix and the cytosol. For simplicity, we consider that a single membrane separates these compartments. Underlying this simplification is the assumption that the IMM poses a much stronger permeation barrier than the OMM, given the abundance of porin/VDAC channels at the latter. Mathematically, this coupling was formalized by adding the permeation terms to the differential equations for the dynamics of cytosolic and matrix H_2O_2 concentrations:

$$\begin{aligned}\frac{d[\mathrm{H_2O_2}]_c}{dt} &= \frac{\kappa A}{V_c}\left([\mathrm{H_2O_2}]_m - [\mathrm{H_2O_2}]_c\right) + \text{remaining terms from Griffith } \textit{et al.} \text{ (2024)}\\ \frac{d[\mathrm{H_2O_2}]_m}{dt} &= \frac{\kappa A}{V_m}\left([\mathrm{H_2O_2}]_c - [\mathrm{H_2O_2}]_m\right) + \text{remaining terms from Stein } \textit{et al.} \text{ (2020)}\end{aligned} \quad (1)$$

with the parameter meanings as indicated in **Table S7**. Here, *K* and *A* stand for the permeability constant and the permeating surface area, respectively. As follows from Equation (1) only the value of the product of these two parameters is relevant for the dynamics. The rationale for the range of *KA* values used in this work is explained in **Method S3**. The reference value given in **Table S7** is close to the lower limit consistent with a PRDX1/2 DKO showing a greater effect on the H_2O_2 concentration in the mitochondrial matrix than a PRDX3/5 DKO, given the values of the other parameters. This value is also close to the best-fit value in the Duong *et al.* (2021) [7] model -- 1.8×10^5 µm^3 s^{-1}, considering the same matrix volume. We adopted a 1000 µm^3 cytosol

volume (V_c), which is typical for human cells, and a 50-fold lower mitochondrial matrix volume (V_m) [8].

To account for the saturability of PRDX3 reduction at high H_2O_2 production rates we replaced the mass action rate expression for TrxR2 in the Stein *et al.* (2020) model by a Michaelis-Menten rate expression for bi-substrate ping-pong mechanism,

$$v_{\mathrm{TrxR2}} = \frac{V_{\mathrm{Max,TrxR2}} \dfrac{[\mathrm{NADPH}]}{K_{\mathrm{M,TrxR2,NADPH}}} \dfrac{[\mathrm{Trx2\text{-}SS}]}{K_{\mathrm{M,TrxR2,Trx2\text{-}SS}}}}{\dfrac{[\mathrm{NADPH}]}{K_{\mathrm{M,TrxR2,NADPH}}} \dfrac{[\mathrm{Trx2\text{-}SS}]}{K_{\mathrm{M,TrxR2,Trx2\text{-}SS}}} + \dfrac{[\mathrm{NADPH}]}{K_{\mathrm{M,TrxR2,NADPH}}} + \dfrac{[\mathrm{Trx2\text{-}SS}]}{K_{\mathrm{M,TrxR2,Trx2\text{-}SS}}}},$$

with the $K_{\mathrm{M,TrxR2,NADPH}}$ and k_{cat} values determined in ref. [9], and $K_{\mathrm{M,TrxR2,TrxR2}}$ assumed as similar to the K_{M} of human TrxR1 for Trx1-SS [10]. We obtained $V_{\mathrm{Max,TrxR2}}$ as the product of k_{cat} by the concentration of TrxR2 in the mitochondrial matrix estimated from a quantitative proteomics dataset for HEK293 cells [11] as described in [12] considering the same matrix volume as above.

We also similarly modified the matrix PRDX3 and PRDX5 concentrations to reflect the same quantitative proteomics dataset [11].

GRX2 has also been reported to reduce oxidized PRDX3 [13]. However, the concentration of GRX2 is lower than that of TRX2; thus, we decided to neglect the impact of GRX2.

To avoid a numerical overflow when modelling a PRDX3 KO, we refined the estimate of the initial H_2O_2 concentration in the mitochondrial matrix in the Stein *et al.* (2020) model by taking the contributions of PRDX5, GPx1, GPx4 and permeation for H_2O_2 clearance into account:

$$[\mathrm{H_2O_2}]_m(0) = \frac{k1}{k6\,\mathrm{Prx3tot} + k2\ \mathrm{GPx1tot} + k23\mathrm{Prx5tot} + k26\mathrm{Prx5tot} + \dfrac{\kappa \mathrm{A}}{V_m}}.$$

Finally, we modified the Griffith et al. (2024) model by considering a ϕ_c= 200 nM s^{-1} basal non-mitochondrial H_2O_2 supply to the cytosol, arising, for instance from peroxisomes, the endoplasmic reticulum and extracellular sources. The rationale for this estimate is presented in SI1 of ref. [6].

The small dimensions of the mitochondrial matrix justify neglecting diffusional restrictions in this compartment. In turn, the neglect of diffusional restrictions in the cytosol is a coarser approximation that leads to a slight overestimation of the cytosolic H_2O_2 clearance activity, especially at higher mitochondrial H_2O_2 release rates. As a consequence, this is a conservative approximation that tends to underestimate the effects of PRDX1 and/or PRDX2 KO on the mitochondrial matrix H_2O_2 concentrations.

Method S2. Calculation of H_2O_2 generation rates

For our modeling approach we assumed a basal H_2O_2 generation flux value of 4 µM s^{-1}, as this was previously assumed to be the flux originating from the respiratory chain in HeLa cells [5]. For the deflection from basal level, we assumed values of 100, 150, 200, and 250 µM s-1 of mitochondria-generated H_2O_2 with 200 µM s-1 calculated to correspond to H_2O_2 generated by mtDAO upon 4 mM D-Ala addition.

Previous reports [14] indicated that in RPE1-hTERT-TOM20−DAO (OMMDAO) cells at 20 mM D-Ala, the H_2O_2 production (inferred from OCR) is about 60 pmol min^{-1} which translates to ~1.5 fmol $cell^{-1}$ min^{-1}. We can assume that D-Ala import into the matrix is limited and thus even lower amounts of D-Ala reach mtDAO in our experimental setting – thus, we would calculate with a maximum rate of ~1 fmol $cell^{-1}$ min^{-1}. Considering a volume of 1000 $µm^3$ per cell, 1 fmol $cell^{-1}$ min^{-1} translates into a ~17 µM s^{-1} H_2O_2 production, and for mtDAO, considering that the volume of matrix is ~1/50 of the cell volume, the local production rate would then be ~833 µM s^{-1}. Supposing that 4 mM D-Ala would lead to ¼ of that production rate, this amounts to ~200 µM s^{-1}.

1 mM D-Ala at which we still observe a clear impact of PRDX1/2 loss on matrix HyPer7 would correspond to about 50 µM s^{-1}, and would reflect for example the ten-fold increase of mitochondrial H_2O_2 generation observed upon antimycin A treatment [1]. To adjust for the delayed onset of DAO-mediated H_2O_2 generation after D-Ala addition that we and others observed, we assumed that H_2O_2 generation would thereby ramp linearly within 5 min to the respective values.

Method S3. Parameter describing IMM transport of H_2O_2

The simulations considered several alternative values of KA, as explained below:

Alternative 1: $A_{IMM} = A_{OMM}$ (assumption $A_{OMM} \approx 0.2 x A_{PM}$ [8]), and K_{IMM} is in the range of K of the plasma membrane (K_{PM}) with 10 µm s^{-1} [12, 15]; this model could be assumed if H_2O_2 homogenously diffuses across the entire IMM, and cristae junctions pose stronger diffusion limits compared to the IMM itself, *i.e.* $[H_2O_2]_{cristae} \approx [H_2O_2]_{matrix}$. → KA ≈ 100 $µm^2$ x 10 µm s^{-1} ≈ 1000 $µm^3$ s^{-1} ≈ **0.01 x 10^5 $µm^3$ s^{-1}**

Alternative 2: $A_{IMM} \approx 10 x A_{OMM}$ (invaginations of IMM), and $K_{IMM} = K_{PM}$; this model could be assumed if H_2O_2 homogenously diffuses across the entire IMM, and cristae junctions pose little diffusion limitation compared to the IMM itself, *i.e.* $[H_2O_2]_{cytosol} = [H_2O_2]_{cristae} < [H_2O_2]_{matrix}$. → KA ≈ 10 x 100 $µm^2$ x 10 µm s^{-1} ≈ **0.1 x 10^5 $µm^3$ s^{-1}.**

Notably, we addressed in a series of reaction-diffusion simulations how effectively cristae junctions could handle H_2O_2. We thereby considered a cylindrical geometry where a cristae junction is a 20 nm diameter, 150 nm long tube surrounded by matrix but opening to the peripheral IMS at one extremity. With such dimensions, even if the permeability constant is just 10 µm s^{-1} virtually any H_2O_2 molecule released from the matrix into the cristae junction will be reabsorbed into the matrix. This process is so fast that it could outcompete 100 µM PRDX, though it is quite unlikely that PRDX1/2 decamers can enter the cristae junctions. It is thus likely that for H_2O_2 release to the

cytosol only the inner boundary membrane directly facing towards the OMM is relevant ($A_{IMM} = A_{OMM}$).

For *Alternative 3*, we calculated which *KA* would be sufficient to explain the stronger effect of the PRDX1/2 DKO than the PRDX3/5 DKO on matrix H_2O_2. This phenomenon is determined by a competition between the H_2O_2 clearance activity in the matrix and H_2O_2 permeation across the IMM. Through a series of simulations of the model described in **Method S1** we determined that *KA* has to be at least one order of magnitude higher than in *Alternative 2* for it to occur, e.g. a *KA* ≈ **1.5 x 10^5 μm^3 s^{-1}**. Assuming that $A_{IMM} = A_{OMM}$ and is thus set, this can only be explained by a K_{IMM} much larger compared to K_{PM}; this could be achieved by dedicated H_2O_2 transporters in the IMM or the richness of the IMM in unsaturated lipids that promotes permeability. A previous study reported on K_{IMM} of isolated rat liver mitochondria in the range of 500 μm s^{-1} [16]. A doubling of *KA* above would yield ≈ **3 x 10^5 μm^3 s^{-1}**, 50-times the value for K_{IMM} assumed in *alternative 1* would yield ≈ **0.5 x 10^5 μm^3 s^{-1}.**

Method S4. Sensitivity assay

To test the robustness of the conclusion that the IMM must be very permeable to H_2O_2 for the PRDX1/2 DKO to have a greater influence on matrix H_2O_2 concentration than the PRDX3/5 DKO, we analyzed the sensitivities of the lowest value of *KA* (KA_{min}) consistent with that outcome to all model parameters. Most parameters turned out to have a negligible influence on KA_{min} and just the following few have a moderate influence. Increasing values of the maximal rate of NADPH regeneration rate, the initial concentration of NADPH in the mitochondrial matrix, the activity of alternative cytosolic H_2O_2 sinks and the activity of TrxR2 in the mitochondrial matrix increase KA_{min} with logarithmic sensitivities 1.2,1.2, 1.0 and 0.54, respectively. These correspond to slightly supra linear to moderately sub linear influences. In turn, increasing values of k_{DAO}, the K_M of NADPH regeneration for $NADP^+$ and the K_M of TrxR2 for NADPH decrease KA_{min} with logarithmic sensitivities -1.4, -0.90 and -0.30, respectively. The lowest KA_{min} value reached by halving the value of any of the former parameters or doubling the value of any of the latter was 3.7×10^4 μm^3 s^{-1}, which is still a very high permeability.

We performed the sensitivity analysis of KA_{min} as follows. For each choice of parameter values we numerically integrated the model up to t= 1 h considering k_{DAO}= 250 μM s^{-1} with a 5 min ramp up as before, to compute the matrix H_2O_2 concentration for each considered *KA* value ($[H_2O_2]_M(KA)$). These numerical integrations were carried out in Mathematica™ v. 14.0.0.0 [*Wolfram Research Inc., Mathematica, Version 14, Wolfram Research, Inc., Champaign, IL, U.S.A., 2024*] using the ParametricNDSolve function with default options. To find KA_{min} we determined the *KA* value that makes $[H_2O_2]_M(KA)$ for the PRDX1/2 DKO identical to that of the PRDX3/5 DKO. For this we used the FindRoot function from Mathematica™ v. 14.0.0.0 with default options. To approximate the logarithmic sensitivity of KA_{min} to a parameter λ

around its reference value in **Table S8** (λ_{ref}), we determined $KA_{min}(\lambda_{ref}/2)$, $KA_{min}(\lambda ref)$ and $KA_{min}(2\lambda_{ref})$, and computed the slope of the log(KA_{min}) vs. log(λ) linear fit based on these three points." These fits resorted to the function Fit from Mathematica^{TM} v. 14.0.0.0 with default options. A logarithmic sensitivity S(KA_{min}, λ) means approximately that a 1% increase in the value of λ causes a S(KA_{min}, λ)% increase (decrease, if negative) in the value of KA_{min}.

Method S5. Preselection of mitochondrial genes, correlated genes from DepMap and aquaporins for the CRISPR knockout library

Gene identifier of mitochondrial genes was integrated from the Gene Ontology (AMIGO 2, 2020-07), the Human MitoCarta2.0 and from The Integrated Mitochondrial Protein Index (IMPI.Q2.2018). Genes and gene products were searched for with help of the AMIGO 2 toolbox using inferred annotation and filters "taxon_subset_closure_label: Homo sapiens" as well as "regulates_closure_label: mitochondrion". The resulting list of UniProtKB identifiers (ids) was exported and the ids were assigned to Ensembl stable ids by using BioMart (GRCh38.p13) [17]. Ensembl stable ids from GO, MitoCarta and IMPI were combined and deprecated ids were removed. For each unique Ensembl stable id of the combined mitochondrial dataset the top five co-dependent genes from the Achilles dataset, i.e. DepMap Public 20Q2, were derived and restricted to only those with a Pearson correlation coefficient cor ≥ 0.3 by a custom R script. Aquaporins neither present in the mitochondrial gene set nor among the correlated genes from DepMap were added to the preselected dataset for the CRISPR knockout library.

Method S6. Design of the sgRNA library

We designed a library of 21.068 sgRNAs targeting exons of 2.802 protein-coding genes in the human genome with a median coverage of 10 sgRNAs per gene. In the exon selection process, individual exons were examined to determine whether specific criteria are met to result in functional knockout of the gene. The exons selected met the following criteria:

(1) Exons containing a translation start or stop signal are omitted, thus, exons containing any UTR sequences are excluded.

(2) Only constitutive exons are selected which are part of all protein-coding transcripts of the target gene.

(3) Primarily exons in the 5' part of the target gene are selected to keep the residual native portion of the original mRNA as short as possible.

(4) Functional protein domains coded on the native coding sequence (CDS) upstream of the selected exon are to be avoided to prevent residual physiological functions in the mutant gene product.

(5) The mutant CDS downstream of the frameshifting mutation should be long enough to enable the translation of ≥ 33 amino acids upstream of the introduced premature stop codon otherwise re-initiation of translation may occur.

(6) Exons overlapping other genes are omitted.

(7) Exons overlapping regulatory features are omitted.

(8) Symmetric exons, i.e. exons flanked by introns of the same phase, are omitted as symmetric exons are thought as putative alternative spliced exons [18].

If exons meeting the above listed criteria have been identified, we used a local installation of the CRISPOR software package [19] to identify highly specific sgRNA sequences in the selected exons having a length of 20 nucleotides and the PAM sequence NGG. On-target specificity as well as the number and genomic location of off-target cut sites are the primary factors affecting the selection of suitable sgRNAs. Several specificity scores have been defined using experimental data [20-23], including the MIT specificity score, for which a cut-off of 66 for sgRNA design is given in the literature [24]. 566.994 raw sgRNAs emitted by CRISPOR are quality filtered as described in detail below:

The sgRNA seed region is defined as the PAM-proximal 10-12 bases of the targeting sequence, which is important for an effective binding of the DNA at the target- or off-target site [25, 26]. Thus, off-targets with mismatches in the guide seed region are ineffectively cut. In addition to specificity, when selecting suitable sgRNAs, care should be taken to ensure that off-target sites of a sgRNA have mismatches solely located in the seed region. We used a filter that classifies each sgRNA according to the number of mismatches in the off-target sites. For this reason, thresholds can be specified for the permissible number of off-target sites with 0, 1, 2 or 3 mismatches, in the total length of the gRNA sequence and specifically in the seed region. Irrespective of their specificity, sgRNAs having off-targets with 0, 1 or 2 mismatches are excluded. Off-target sites with 3 or more mismatches are not effectively cut by the Cas9 enzyme [27] and such sgRNAs are therefore selected as potential candidates for gene editing. sgRNAs with specific T-rich sequence motifs within the seed region that impede pairing with the target DNA [28] are excluded. Non-isogenicity between the reference sequence of the target gene, which is used for gRNA finding, and the genome sequence of the cellular material used in the screen can substantially influence the pairing efficiency of sgRNAs [29]. sgRNAs overlapping cell-type-specific sequence variations (HEK293T) are therefore excluded.

To find enough sgRNAs according to our criteria, we used a 3-step approach:

If a significant number of highly specific sgRNAs with a MIT specificity score of >80 cannot be found in the exon sequence, the filter process is repeated for this exon with

a MIT score between 60 and 80. Accordingly, we relaxed the number of mismatches allowed up to 200 off-targets with 3 mismatches, up to 50 off-targets with 2 mismatches, and up to 10 off-targets with 1 mismatch, while sgRNAs with off-targets without any mismatch are excluded. If still not enough sgRNAs are identified for the target exon, the filter is reduced to an MIT score of 50 to 60. Finally, the first base of the sgRNA was substituted to a G to improve sgRNA expression [30]. Gene structure data, genomic and mRNA sequences, genome feature and sequence variation data from Ensembl (genome build GRCh38) are used for the analysis [31].

Pre-designed mitochondria-focused sgRNAs were cloned into the lentiviral CRISPY backbone. Briefly, the pCDH-EF1-CRISPY vector was generated by replacing the EF-1 promoter, multiple cloning site, and WPRE of the parental pCDH-EF1 vector with a synthetic cassette containing a human U6 promoter with BbsI cloning sites, a chimeric sgRNA scaffold, and an EF1α-driven SpCas9 fused to a triple NLS and FLAG tag, followed by a P2A-linked puromycin resistance gene and a minimal WPRE. The sgRNA library was inserted via BbsI cloning. sgRNA sequences are listed in the **Supplementary dataset 2**.

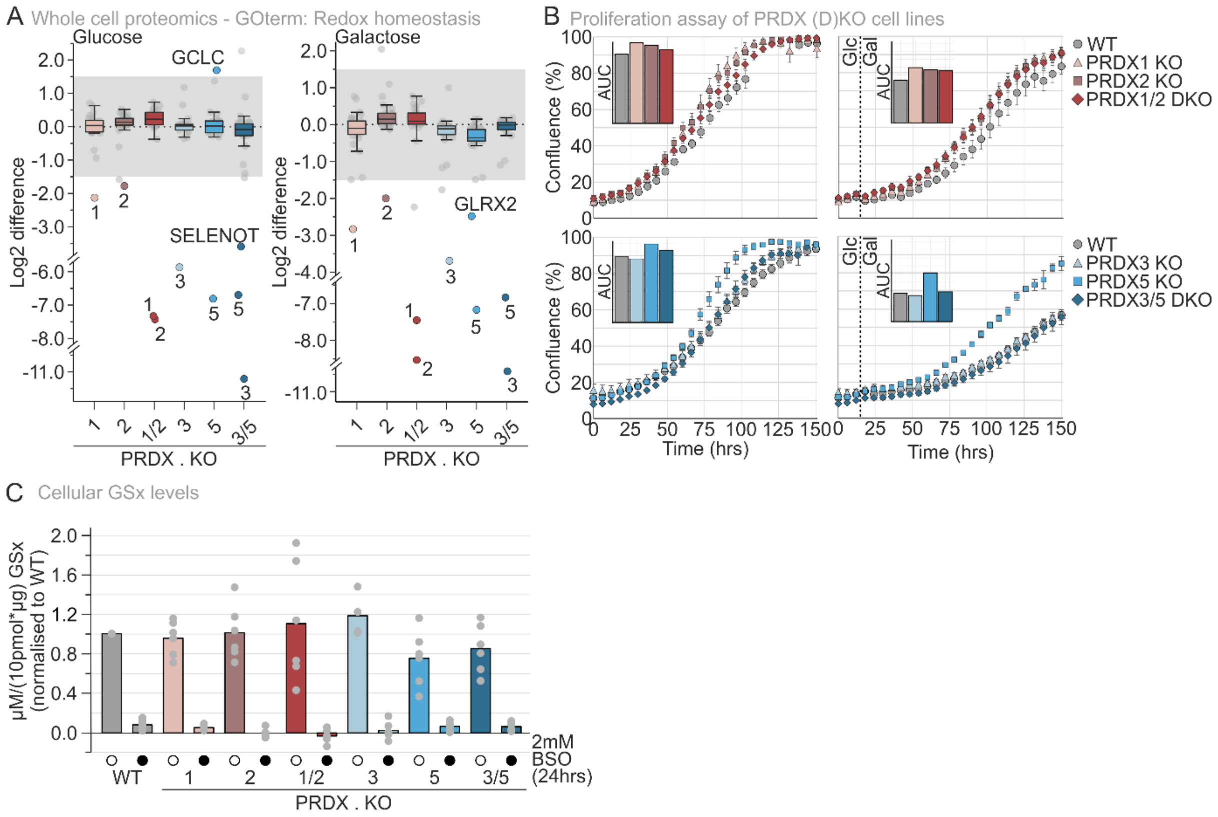

Figure S1. Characterization of PRDX KO cell lines.

(A) Go term analysis, involving redox homeostasis, of quantitative proteomics (n = 4 replicates). Visualized via boxplot difference, in which the boxplot visualizes the median, 1st and 3rd quartile and the whiskers 1.5 × IQR. Colored and labeled circles are hits which were significant (LFC -1.5 < or >1.5 and p ≤ 0.05). Cells were grown on glucose and adapted for minimal two weeks on galactose. Interestingly, the consistently decreased HyPer7 signal in the PRDX5 KO at BL (**Figure 1C, 2B**) might reflect increased reductive capacity related to the adaptive GCLC upregulation that we observed in those cells.

(B) Assessment of proliferation of wild type, and the generated KO cell lines. Cytosolic PRDX KO cell lines are represented on top, and the mitochondrial KO cell lines are represented in the bottom. Growth was assessed on glucose as well as upon galactose shift. AUC is represented as bar diagram in which the seeding density was subtracted.

(C) Total cellular glutathione content (GSx) was measured via a recycling assay. The bar diagram visualizes the mean of six experimental replicates. Significance was assessed via the Student's t-test.

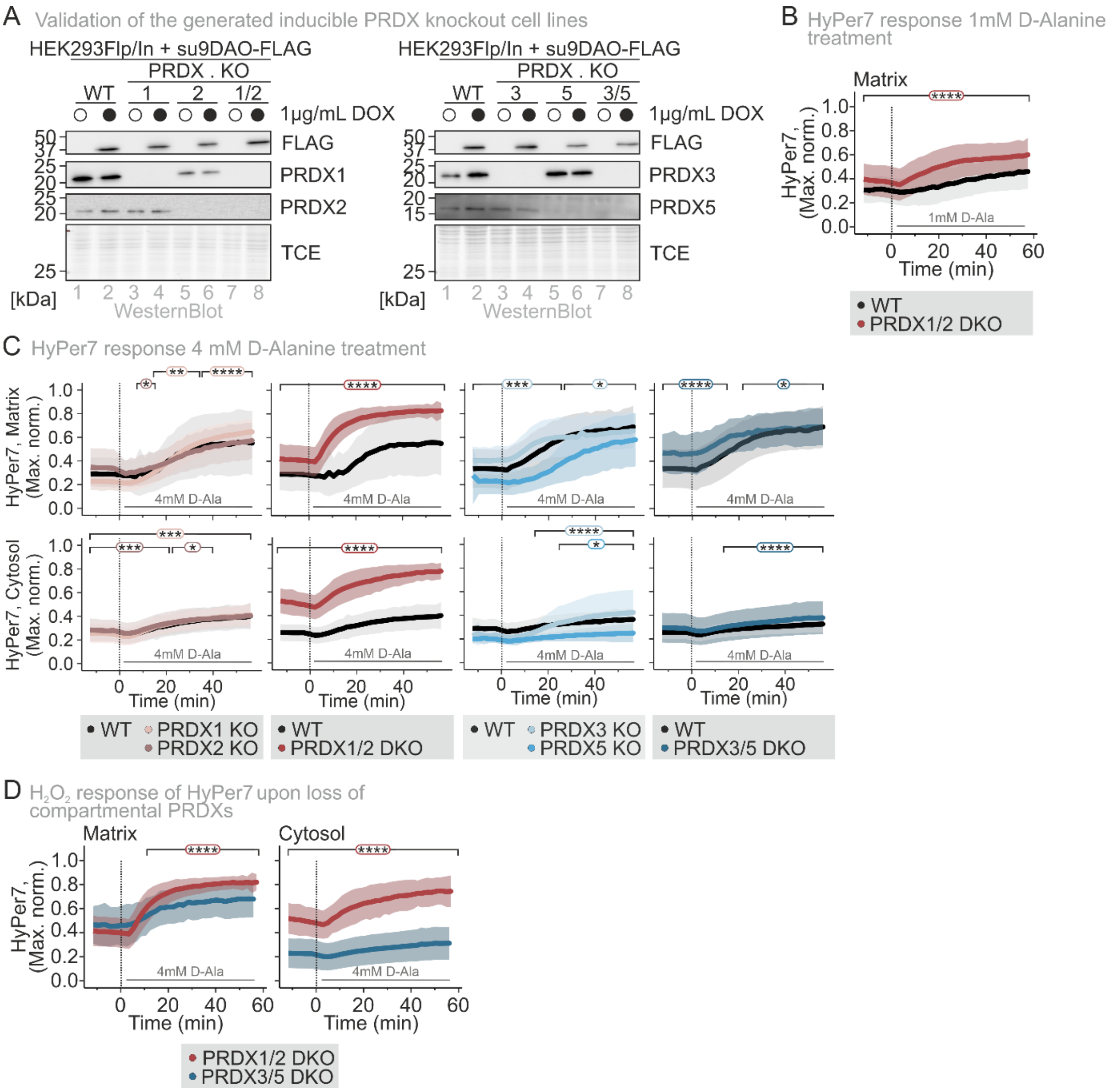

Figure S2. Mitochondrial DAO cell lines.

(A) Peroxiredoxin levels in the generated single (KO) and double (DKO) knockout cell lines expressing the mtDAO-FLAG. Immunoblot analysis of lysates of the generated KO/DKO cell lines, with an inducible expression of mtDAO by addition of doxycycline (DOX).

(B) Response of matrix HyPer7 upon treatment with 1 mM D-Alanine. Cells were grown in glucose-containing medium. The number of cells, number of experimental replicates and information on statistical analysis and visualization can be found in **Supplementary dataset S1**.

(C) Response of HyPer7 in the generated cell lines expressing mtDAO upon treatment with 4 mM D-Alanine (black, WT; pink, PRDX1 KO; brown, PRDX2 KO; dark red, PRDX1/2 DKO; light blue, PRDX3 KO; blue, PRDX5 KO; dark blue, PRDX3/5 DKO). HyPer7 sensors were targeted to the indicated subcellular compartments. The cells were grown in glucose-containing medium. Solid line represents average, and the standard deviation is represented by the

background in the respective color. The number of cells, number of experimental replicates and information on statistical analysis and visualization can be found in **Supplementary dataset 1**.

(D) Response of HyPer7 in PRDX DKO cell lines expressing mtDAO upon treatment with 4 mM D-Alanine (dark red, PRDX1/2 DKO; dark blue, PRDX3/5 DKO). HyPer7 sensors were targeted to the indicated subcellular compartments. Cells were grown in glucose-containing medium. The number of cells, the number of experimental replicates, and information on statistical analysis and visualization can be found in **Supplementary dataset 1**.

* P ≤ 0.05, ** P ≤ 0.01, *** P ≤ 0.001, **** P ≤ 0.0001.

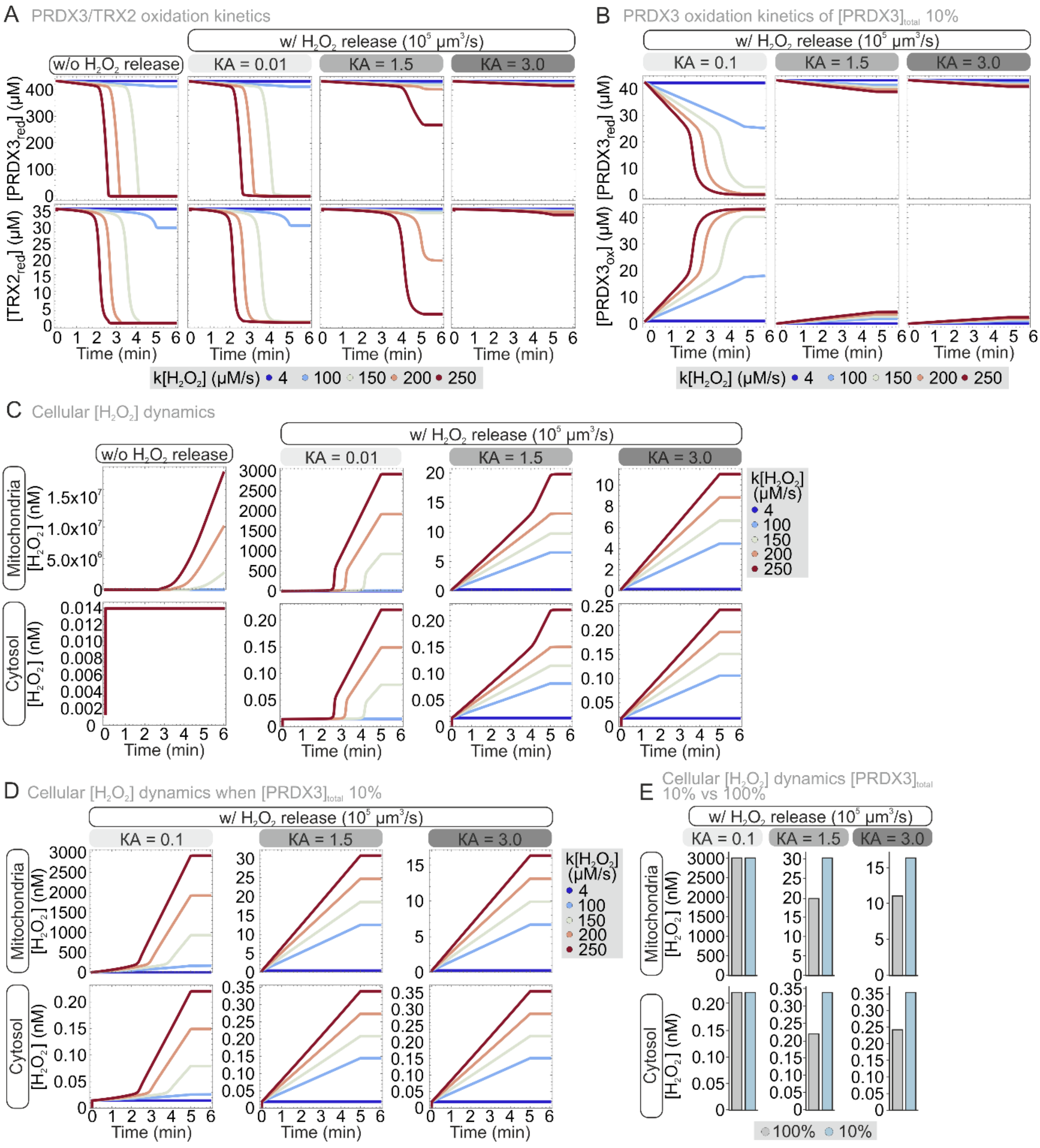
A PRDX3/TRX2 oxidation kinetics
w/ H2O2 release (10^5 μm^3/s)
w/o H2O2 release
KA = 0.01
KA = 1.5
KA = 3.0
[PRDX3red] (μM)
[TRX2red] (μM)
Time (min)
k[H2O2] (μM/s) • 4 • 100 • 150 • 200 • 250
B PRDX3 oxidation kinetics of [PRDX3]total 10%
w/ H2O2 release (10^5 μm^3/s)
KA = 0.1
KA = 1.5
KA = 3.0
[PRDX3red] (μM)
[PRDX3ox] (μM)
Time (min)
k[H2O2] (μM/s) • 4 • 100 • 150 • 200 • 250
C Cellular [H2O2] dynamics
w/ H2O2 release (10^5 μm^3/s)
w/o H2O2 release
KA = 0.01
KA = 1.5
KA = 3.0
Mitochondria [H2O2] (nM)
Cytosol [H2O2] (nM)
Time (min)
k[H2O2] (μM/s) • 4 • 100 • 150 • 200 • 250
D Cellular [H2O2] dynamics when [PRDX3]total 10%
w/ H2O2 release (10^5 μm^3/s)
KA = 0.1
KA = 1.5
KA = 3.0
Mitochondria [H2O2] (nM)
Cytosol [H2O2] (nM)
Time (min)
k[H2O2] (μM/s) • 4 • 100 • 150 • 200 • 250
E Cellular [H2O2] dynamics [PRDX3]total 10% vs 100%
w/ H2O2 release (10^5 μm^3/s)
KA = 0.1
KA = 1.5
KA = 3.0
Mitochondria [H2O2] (nM)
Cytosol [H2O2] (nM)
• 100% • 10%

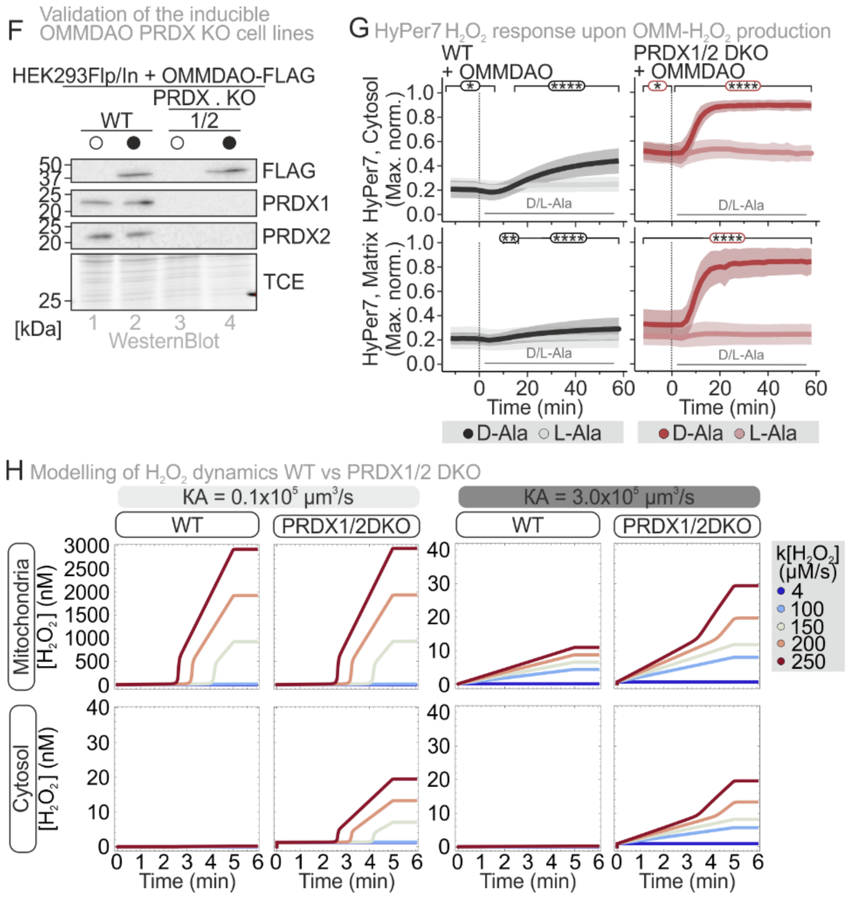

Figure S3. Modelling cellular H_2O_2 dynamics.

(A) Behavior of the reduced PRDX3 and TRX2 species upon continuous generation of matrix H_2O_2 (k[H_2O_2] (µM s-1)) with and without H_2O_2 release (ΚA = 0 and ΚA = 0.01 – 3.0 x10^5 µm^3 s^{-1}).

(B) Behavior of reduced and oxidized PRDX3 species upon lowered PRDX3 [10%] levels. The behavior was modeled upon continuous generation of matrix H_2O_2 (k[H_2O_2] (µM s^{-1})) with increased H_2O_2 release (ΚA = 0.01 – 3.0 x10^5 µm^3 s^{-1}).

(C) Cellular H_2O_2 dynamics upon increased mitochondrial H_2O_2 production (k[H_2O_2] (µM s^{-1})) with and without H_2O_2 release (ΚA = 0 and ΚA = 0.01 – 3.0 x10^5 µm^3 s^{-1}).

(D) Cellular H_2O_2 dynamics of $[PRDX3]_{total}$ 10% upon increased mitochondrial H_2O_2 production (k[H_2O_2] (µM s^{-1})) with increased H_2O_2 release (ΚA = 0.01 – 3.0 x10^5 µm^3 s^{-1}).

(E) Comparison of the max [H_2O_2] upon mitochondrial H_2O_2 generation of 250 µM s^{-1} when [PRDX3] is 100% vs 10%.

(F) Peroxiredoxin levels in the generated single (KO) and double knockout (DKO) cell lines expressing the OMM-DAO-FLAG. Immunoblot analysis of lysates of the generated knockout cell lines, with an inducible expression of OMM-DAO by addition of doxycycline.

(G) Response of HyPer7 in PRDX1/2 DKO cell line expressing OMMDAO upon treatment with 8 mM D-Alanine (dark red, PRDX1/2 DKO; black WT) and 16 mM

L-Alanine (pink, PRDX1/2DKO; grey, WT). HyPer7 sensors were targeted to the indicated subcellular compartments. Cells were grown in glucose-containing medium. The number of cells, the number of experimental replicates, and information on statistical analysis and visualization can be found in **Supplementary dataset 1**.

(H) Cellular H_2O_2 dynamics upon loss of PRDX1 and PRDX2, upon increased mitochondrial H_2O_2 production (k[H_2O_2] 4 and 250 µM s^{-1}) with H_2O_2 release (ΚA = 0.01 and 3.0 x10^5 $µm^3$ s^{-1}).

* P ≤ 0.05, ** P ≤ 0.01, **** P ≤ 0.0001.

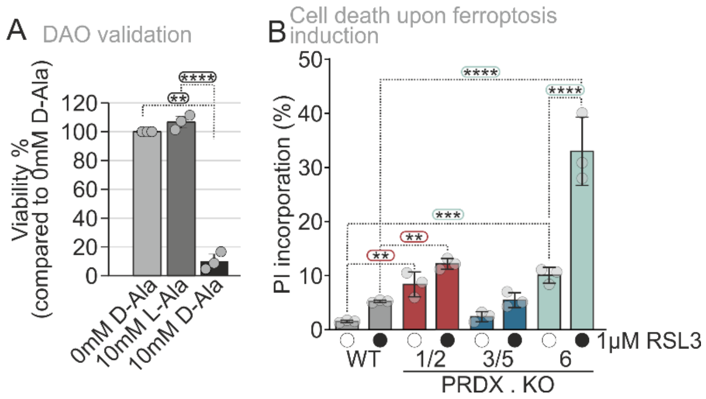

Figure S4. Viability and ferroptosis analysis of PRDX KO cell lines.

(A) Viability of wildtype (WT) cells expressing mt-DAO was assessed upon treatment with 10 mM D/L-Alanine. The cells were grown in glucose-containing medium. The bar diagram represents the average over three experimental replicates each with eight technical replicates, and the standard deviation is represented by the whiskers. Significance was assessed using the Welch's t-test.

(B) Ferroptosis assay indicated no significant influence of the 2-Cys PRDXs double knockouts on ferroptosis. Cells were treated with different concentrations of RSL3, a ferroptosis trigger, where after incorporation of propidium iodide (PI) was measured by FACS analysis. Significance was assessed using the 2-way ANOVA test.

** $P \leq 0.01$, *** $P \leq 0.001$, **** $P \leq 0.0001$.

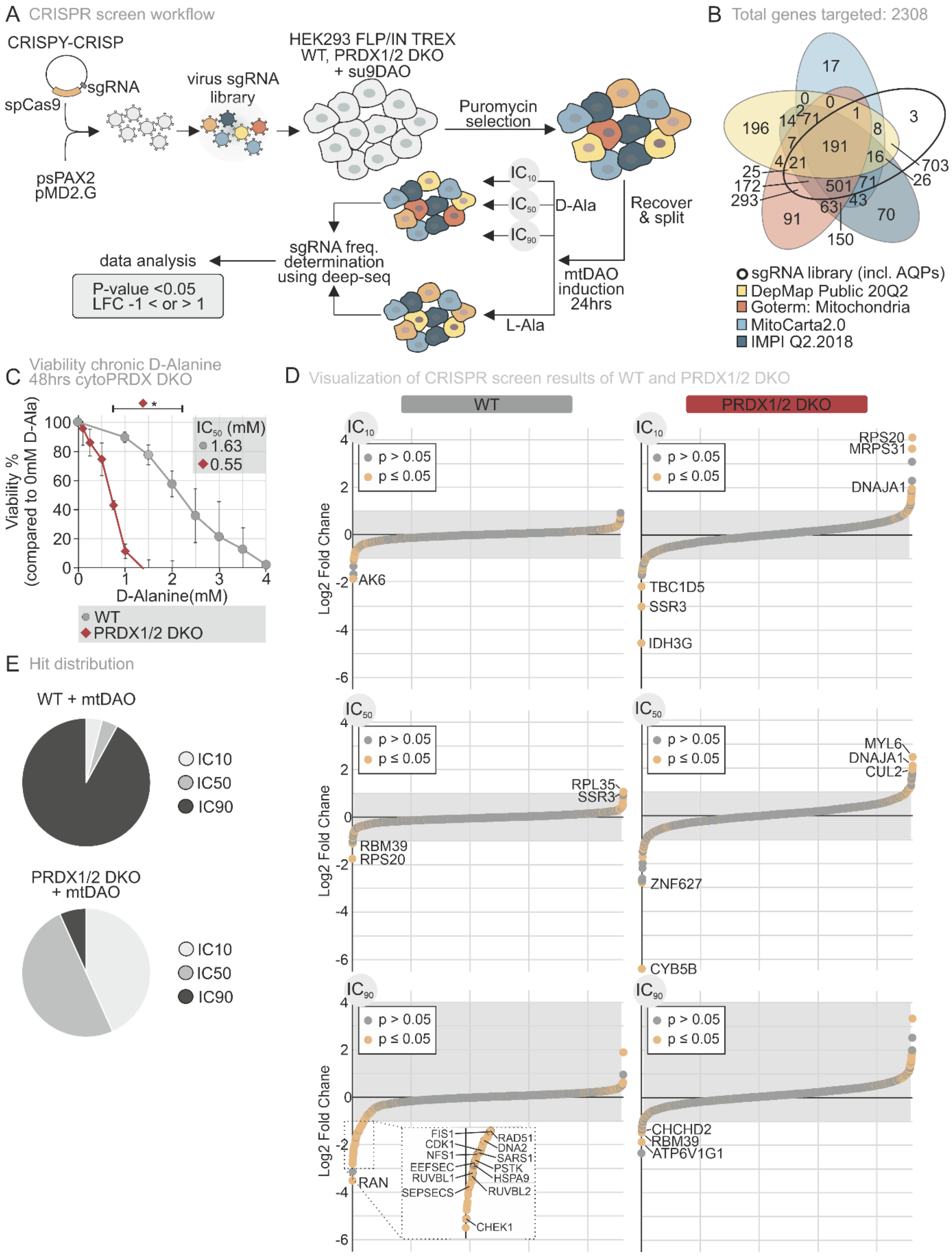
A CRISPR screen workflow
CRISPY-CRISP
sgRNA
spCas9
psPAX2
pMD2.G
virus sgRNA library
HEK293 FLP/IN TREX WT, PRDX1/2 DKO + su9DAO
Puromycin selection
Recover & split
D-Ala
L-Ala
mtDAO induction 24hrs
sgRNA freq. determination using deep-seq
data analysis
P-value <0.05
LFC -1 < or > 1
B Total genes targeted: 2308
sgRNA library (incl. AQPs)
DepMap Public 20Q2
Goterm: Mitochondria
MitoCarta2.0
IMPI Q2.2018
C Viability chronic D-Alanine 48hrs cytoPRDX DKO
Viability % (compared to 0mM D-Ala)
D-Alanine(mM)
IC50 (mM)
1.63
0.55
WT
PRDX1/2 DKO
D Visualization of CRISPR screen results of WT and PRDX1/2 DKO
WT
PRDX1/2 DKO
Log2 Fold Chane
p > 0.05
p ≤ 0.05
AK6
RPS20
MRPS31
DNAJA1
TBC1D5
SSR3
IDH3G
RPL35
SSR3
RBM39
RPS20
MYL6
DNAJA1
CUL2
ZNF627
CYB5B
FIS1
CDK1
NFS1
EEFSEC
RUVBL1
SEPSECS
RAD51
DNA2
SARS1
PSTK
HSPA9
RUVBL2
CHEK1
RAN
CHCHD2
RBM39
ATP6V1G1
E Hit distribution
WT + mtDAO
PRDX1/2 DKO + mtDAO
IC10
IC50
IC90

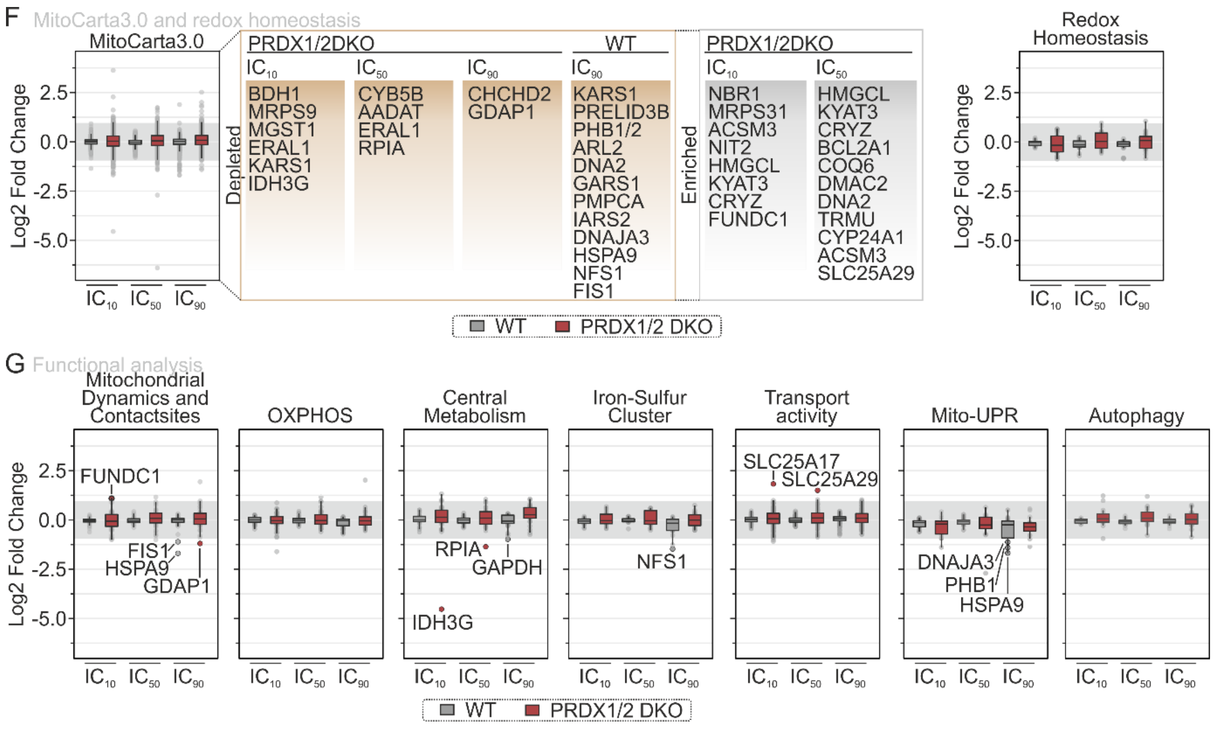

Figure S5. Targeted CRISPR drop out screen.

(A) CRISPR/Cas9 drop-out screen workflow, in which the CRISPR/Cas9 vector was virally transduced in wild type (WT) and PRDX1/2 DKO cell lines expressing the mtDAO system. Three different chronic H_2O_2 stresses were tested by adding various concentrations of D-Alanine. The sequencing data was analyzed via MAGeCK, and measured sgRNAs were deemed hits when they met the set thresholds of (i) a p-value ≤ 0.05, and (ii) LFC of -1 < or > 1.

(B) Set up of the sgRNA library with the use of four databases: (i) GO, (ii) the human mitocarta 2.0, (iii) the IMPI (Q2.2018), and (iv) DepMap 20Q2.

(C) Viability of wildtype (WT) and PRDX1/2 DKO cells was assessed upon treatment with D/L-Alanine for 48 hours. The cells were grown in glucose-containing medium. The bar diagram represents the average over three experimental replicates each with eight technical replicates, and the standard deviation is represented by the whiskers. Significance was assessed using the Welch's t-test.

(D) Visualization of the obtained results for the WT (*left*) and the PRDX1/2 DKO (*right*) of each condition (top, IC_{10}; middle, IC_{50}; bottom, IC_{90}). Hits were deemed significant if the p-value ≤ 0.05 (dark blue) and had an LFC of -1 < or >1 (grey box).

(E) Pie chart to visualize the hit distribution across the three different conditions for the WT and PRDX1/2 DKO cell lines.

(F) MitoCarta3.0 association was visualized via boxplot LFC (*left*), in which the boxplot visualizes the median, 1st and 3rd quartiles, and the whiskers 1.5 × IQR. Colored and mentioned circles are hits which were significant (LFC -1 < or >1 and p ≤ 0.05). RRAscore (*right*), and the sgRNA count (*bottom*) of hits that prove the functionality of the screen in the wild-type cell line expressing the mtDAO.

(G) Functional characterization via GO term analysis, involving redox homeostasis, mitochondrial dynamics and contact sites, OXPHOS, central metabolism, iron-sulfur cluster, and transport activity. Visualized via boxplot LFC (*left*), in which the boxplot visualizes the median, 1st and 3rd quartiles, and the whiskers 1.5 × IQR. Colored and mentioned circles are hits which were significant (LFC -1 < or >1 and p ≤ 0.05). RRAscore (*right*), and the sgRNA count (*bottom*) of hits that prove the functionality of the screen in the wild-type cell line expressing the mtDAO.

* P ≤ 0.05.

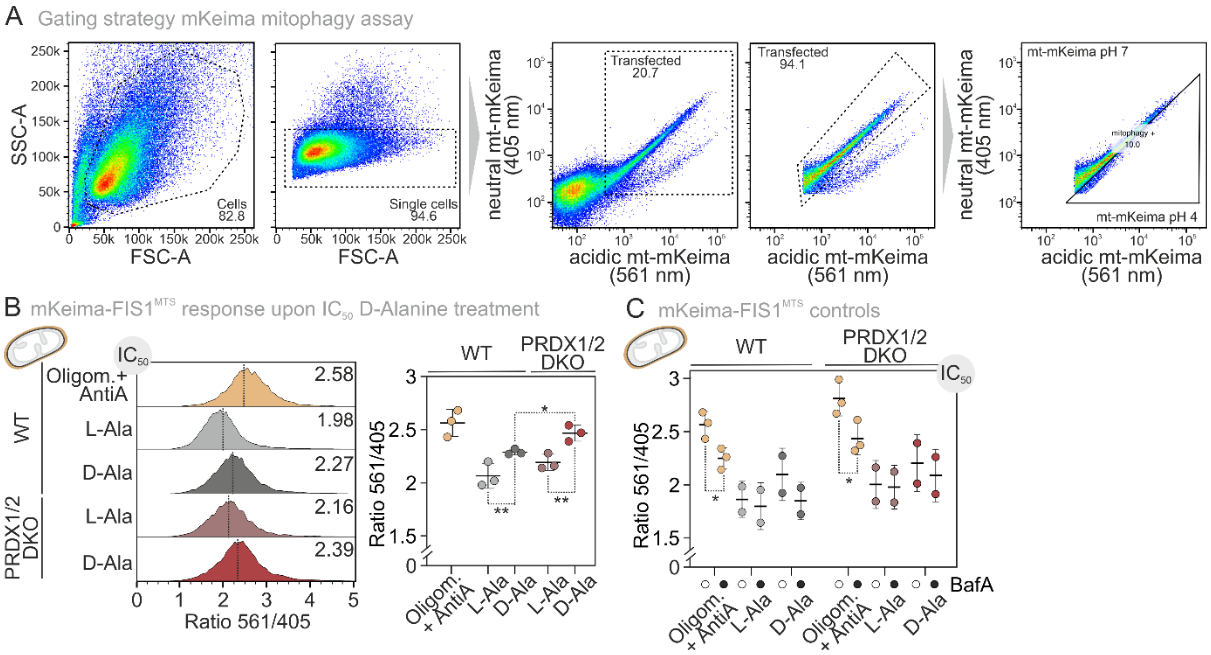

Figure S6. Investigation of mitophagy.

(A) Gating strategy to analyse mKeima signals.

(B) FACS results of steady state mKeima-FIS1^{MTS} in wild type (WT, grey) and PRDX1/2 DKO (dark red) cell lines expressing the mtDAO system, when treated with D/L-Alanine for 8 hours. The histogram (*left*) is a representative FACS result of three experimental replicates. The scatter plot (*right*) represents these three experimental replicates, visualizing the mean and standard deviation. Significance was assessed using the two-way ANOVA test.

(C) FACS results of steady state mKeima-FIS1^{MTS} in wild type (WT, grey) and PRDX1/2 DKO (dark red) cell lines expressing the mtDAO system. Control treatments included a combination of oligomycin and antimycin, with or without bafilomycin A. The scatter plot represents two experimental replicates, visualizing the mean and standard deviation. Significance was assessed using the two-way ANOVA test.

References

1. Hoehne, M.N., et al., Spatial and temporal control of mitochondrial H2 O2 release in intact human cells, EMBO J, 2022. **41**(7): p. e109169.
2. Scherschel, M., et al., A family of NADPH/NADP(+) biosensors reveals in vivo dynamics of central redox metabolism across eukaryotes, Nat Commun, 2024. **15**(1): p. 10704.
3. Pak, V.V., et al., Ultrasensitive Genetically Encoded Indicator for Hydrogen Peroxide Identifies Roles for the Oxidant in Cell Migration and Mitochondrial Function, Cell Metab, 2020. **31**(3): p. 642-653 e6.
4. Fricker, M.D., Quantitative Redox Imaging Software, Antioxid Redox Signal, 2016. **24**(13): p. 752-62.
5. Stein, K.T., et al., Kinetic modeling of H2O2 dynamics in the mitochondria of HeLa cells, PLoS Comput Biol, 2020. **16**(9): p. e1008202.
6. Griffith, M., et al., The architecture of redox microdomains: Cascading gradients and peroxiredoxins' redox-oligomeric coupling integrate redox signaling and antioxidant protection, Redox Biol, 2024. **69**: p. 103000.
7. Duong, Q.V., et al., Identifying Site-Specific Superoxide and Hydrogen Peroxide Production Rates From the Mitochondrial Electron Transport System Using a Computational Strategy, Function (Oxf), 2021. **2**(6): p. zqab050.
8. Hendriks, K.D.W., et al., Differences in mitochondrial function and morphology during cooling and rewarming between hibernator and non-hibernator derived kidney epithelial cells, Sci Rep, 2017. **7**(1): p. 15482.
9. Miseviciene, L., et al., The comparative study of redox properties of recombinant human cytosolic and mitochondrial NADPH:thioredoxin reductases, Chemija, 2022. **33**(2): p. 40-45.
10. Turanov, A.A., et al., Characterization of alternative cytosolic forms and cellular targets of mouse mitochondrial thioredoxin reductase, J Biol Chem, 2006. **281**(32): p. 22953-63.
11. Geiger, T., et al., Comparative proteomic analysis of eleven common cell lines reveals ubiquitous but varying expression of most proteins, Mol Cell Proteomics, 2012. **11**(3): p. M111 014050.
12. Selvaggio, G., et al., Mapping the phenotypic repertoire of the cytoplasmic 2-Cys peroxiredoxin - Thioredoxin system. 1. Understanding commonalities and differences among cell types, Redox Biol, 2018. **15**: p. 297-315.
13. Hanschmann, E.M., et al., Both thioredoxin 2 and glutaredoxin 2 contribute to the reduction of the mitochondrial 2-Cys peroxiredoxin Prx3, J Biol Chem, 2010. **285**(52): p. 40699-705.
14. den Toom, W.T.F., et al., Oxygen-consumption based quantification of chemogenetic H(2)O(2) production in live human cells, Free Radic Biol Med, 2023. **206**: p. 134-142.
15. Moller, M.N., et al., Diffusion and Transport of Reactive Species Across Cell Membranes, Adv Exp Med Biol, 2019. **1127**: p. 3-19.
16. Calamita, G., et al., The inner mitochondrial membrane has aquaporin-8 water channels and is highly permeable to water, J Biol Chem, 2005. **280**(17): p. 17149-53.
17. Smedley, D., et al., The BioMart community portal: an innovative alternative to large, centralized data repositories, Nucleic Acids Res, 2015. **43**(W1): p. W589-98.

18. Schad, E., et al., Exon-phase symmetry and intrinsic structural disorder promote modular evolution in the human genome, Nucleic Acids Res, 2013. **41**(8): p. 4409-22.
19. Haeussler, M., et al., Evaluation of off-target and on-target scoring algorithms and integration into the guide RNA selection tool CRISPOR, Genome Biol, 2016. **17**(1): p. 148.
20. Stemmer, M., et al., CCTop: An Intuitive, Flexible and Reliable CRISPR/Cas9 Target Prediction Tool, PLoS One, 2015. **10**(4): p. e0124633.
21. Singh, R., et al., Cas9-chromatin binding information enables more accurate CRISPR off-target prediction, Nucleic Acids Res, 2015. **43**(18): p. e118.
22. Hsu, P.D., et al., DNA targeting specificity of RNA-guided Cas9 nucleases, Nat Biotechnol, 2013. **31**(9): p. 827-32.
23. Doench, J.G., et al., Optimized sgRNA design to maximize activity and minimize off-target effects of CRISPR-Cas9, Nat Biotechnol, 2016. **34**(2): p. 184-191.
24. Anderson, K.R., et al., CRISPR off-target analysis in genetically engineered rats and mice, Nat Methods, 2018. **15**(7): p. 512-514.
25. Jinek, M., et al., A programmable dual-RNA-guided DNA endonuclease in adaptive bacterial immunity, Science, 2012. **337**(6096): p. 816-21.
26. Kuscu, C., et al., Genome-wide analysis reveals characteristics of off-target sites bound by the Cas9 endonuclease, Nat Biotechnol, 2014. **32**(7): p. 677-83.
27. Anderson, E.M., et al., Systematic analysis of CRISPR-Cas9 mismatch tolerance reveals low levels of off-target activity, J Biotechnol, 2015. **211**: p. 56-65.
28. Graf, R., et al., sgRNA Sequence Motifs Blocking Efficient CRISPR/Cas9-Mediated Gene Editing, Cell Rep, 2019. **26**(5): p. 1098-1103 e3.
29. Andreasson, C., et al., Direct cloning of isogenic murine DNA in yeast and relevance of isogenicity for targeting in embryonic stem cells, PLoS One, 2013. **8**(9): p. e74207.
30. Koike-Yusa, H., et al., Genome-wide recessive genetic screening in mammalian cells with a lentiviral CRISPR-guide RNA library, Nat Biotechnol, 2014. **32**(3): p. 267-73.
31. Zerbino, D.R., et al., Ensembl 2018, Nucleic Acids Res, 2018. **46**(D1): p. D754-D761.
